# 2-Styrylquinolines with Push-Pull Architectures as Sensors for β-Amyloid Aggregation with Theranostic Properties

**DOI:** 10.3390/ijms26178270

**Published:** 2025-08-26

**Authors:** Marta Piquero, Álvaro Sarabia-Vallejo, Latoya Bote-Matías, Gonzalo León-Espinosa, Macarena Hernández-Arasti, Sagrario Martín-Aragón, Paloma Bermejo-Bescós, Ana I. Olives, Pilar López-Alvarado, M. Antonia Martín, J. Carlos Menéndez

**Affiliations:** 1 Unidad de Química Orgánica y Farmacéutica, Departamento de Química en Ciencias Farmacéuticas, Facultad de Farmacia, Universidad Complutense, Plaza de Ramón y Cajal s/n, 28040 Madrid, Spain; mpiqueromarti@gmail.com (M.P.); alsarabi@ucm.es (Á.S.-V.); alvarado@ucm.es (P.L.-A.); 2 Unidad de Química Analítica, Departamento de Química en Ciencias Farmacéuticas, Facultad de Farmacia, Universidad Complutense, Plaza de Ramón y Cajal s/n, 28040 Madrid, Spain; ariesgen@gmail.com (L.B.-M.); aiolives@ucm.es (A.I.O.); mantonia@farm.ucm.es (M.A.M.); 3 Departamento de Química y Bioquímica, Facultad de Farmacia, Universidad San Pablo-CEU, CEU Universities, Urbanización Montepríncipe, Boadilla del Monte, 28660 Madrid, Spain; gonzalo.leonespinosa@ceu.es; 4 Departamento de Farmacología, Farmacognosia y Botánica, Facultad de Farmacia, Universidad Complutense, Plaza de Ramón y Cajal s/n, 28040 Madrid, Spain; macarenaarasti@gmail.com (M.H.-A.); smartina@ucm.es (S.M.-A.); bescos@ucm.es (P.B.-B.)

**Keywords:** fluorescence sensors, proteinopathies, beta-amyloid aggregates, Alzheimer’s disease, neuroprotection, theranostics

## Abstract

The design and synthesis of a small library of 2-styrylquinoline derivatives containing a push-pull system, aimed at displacing their fluorescence emission towards the NIR region, is described. We describe here their synthesis, fluorescent characterization and pharmacological evaluation against different amyloid proteins. Their study showed that these compounds are capable to change their spectroscopic properties upon protein interaction, resulting in changes in the absorption and emission wavelengths, together with increased fluorescence intensity. They also showed sensitivity to pH and environment polarity, exhibiting red shifts in lower polarity environments with regard to aqueous media. Inner charge transfer is observed and employed for detecting the interaction of these compounds with protein aggregates. The study of the alterations in the fluorescence intensity allows to calculate the dissociation constant values for the protein-sensor interaction. These spectroscopic results were the basis for the use of these compounds to visualize β-amyloid plates with selectivity over phosphorylated tau in samples of cerebral tissue from deceased Alzheimer patients under fluorescence microscopy, using immunofluorescence techniques. Pharmacological assays showed that the compounds inhibit the aggregation of the Aβ_1–42_ and AcPHF6 peptides, representing tau protein. They also showed neuroprotective activity following okadaic acid insult.

## 1. Introduction

Proteinopathies encompass a diverse group of disorders caused by the abnormal accumulation and aggregation of specific proteins within cells or tissues. This category includes numerous human diseases, particularly neurodegenerative conditions such as Alzheimer’s, Parkinson’s and prion diseases and amyotrophic lateral sclerosis (ALS) [1], as well as other systemic disorders like transthyretin amyloidosis and some aspects of type II diabetes. The global impact of these diseases is immense and is on the rise due to the increase in life expectancy. Alzheimer’s disease, the leading cause of dementia, currently affects over 55 million people, with projections estimating that this number will triple by 2050, posing significant challenges to healthcare systems globally [2,3]. In the case of Parkinson’s, the second most prevalent neurodegenerative disease, the incidence is 10–20/100,000 [4], with an estimated current number of cases around 12 million worldwide, with a projection of 25 million cases by 2050 [5]. Other proteinopathies are less prevalent (prion diseases 1–2/1,000,000 [6]; ALS 0.7–3.8/100,000 [7]; transthyretin amyloidosis 18–55/100,000 [8]), but they still have huge social impacts [9].

Proteinopathies generally arise from protein misfolding and are thus known as protein misfolding diseases (PMDs). In the course of these diseases, a conformational change of normally expressed proteins takes place that involves their transformation from a physiological soluble monomeric form into oligomeric and fibrillary forms, rich in proteolysis-stable, β-sheet regions. The transformation from normal to abnormal forms is intimately linked to oxidative stress processes linked to cellular aging [10] and is particularly relevant in neurodegenerative diseases [11,12]. In addition to the proteins capable of forming insoluble aggregates located in the central nervous system, there are many other proteins with amyloidogenic character such as lysozyme [13], apolipoprotein [14], amylin [15] and insulin [16], among others [17]. The interrogation of such amyloid aggregates using small molecules is a particularly relevant area of research from both analytical [18] and therapeutic [19] perspectives.

Alzheimer’s disease (AD) is characterized by extracellular amyloid plaques formed by misfolded Aβ peptide. Despite the lack of sequence homology between the corresponding proteins, aromatic residues are recognized as common key motifs in the formation and stabilization of amyloid structures via π-π stacking. Thus, targeting aromatic recognition interfaces could be a useful approach for inhibiting amyloid formation as well as disrupting the preformed amyloid fibrils [20]. Another protein that plays a key role in AD etiopathology is tau. Under physiological conditions, it regulates the stability and assembly of microtubules, affects synaptic integrity and axonal transport and it also participates in protein trafficking and signaling [21]. The phosphorylation levels of tau are regulated by specific enzymes such as GSK3β, PKA, PP2A, and CDK5, which thus control microtubule function. In Alzheimer’s disease patients, impairment in the activity of some of these enzymes leads to elevated levels of phosphorylated tau. This protein is prone to aggregation, forming helical filaments that ultimately give rise to neurofibrillary tangles. Their accumulation disrupts microtubule assembly and results neuronal death [22]. Additional deficiencies found in AD include calcium homeostasis deregulation, mitochondrial dysfunction, oxidative stress, neuroinflammation and deterioration of neurotransmission, particularly at cholinergic neurons.

The diagnosis of Alzheimer’s disease is mainly based on physical, cognitive, and neurobiological assessment or is performed *post-mortem* by using misfolded proteins as neuropathological hallmarks. Since fibrillary aggregates are believed to appear many years before clinical symptoms, imaging of these aggregates is particularly adequate for the early diagnosis of the onset of the disease, which is crucial for successful therapy, and monitor its progression. In the initial stage of AD, taking place 5–10 years before dementia diagnosis, a sharp increase in Aβ_42_ levels can be detected, while phospho-tau levels in cerebrospinal fluid rise more gradually [23,24]. The increases in Aβ_42_ and tau levels are early events that precede atrophy, neurodegeneration and clinical symptoms [25]. Therefore, developing new methodologies that enable the early visualization of Aβ_42_ aggregates represents a promising strategy for the early diagnosis of Alzheimer’s disease. In this context, optical imaging using fluorescence appears as a highly promising technique with a high sensitivity and that does not involve the use of radioactivity and has advantages such as good sensitivity, low cost, easy operation, capability of real-time monitoring and very low toxicity and exposition [26,27,28]. The emergence of near infrared (NIR) imaging has provided an effective, non-invasive technique for visualizing amyloid plaques in vivo. The detection of fluorescence emission in the NIR region has considerable advantages, given that the background signal due to tissue fluids, caused mainly by the intrinsic fluorescence of proteins, occurs in the ultraviolet region of the spectrum, although some biomolecules present in cells can emit in the visible region, normally in the 400–450 nm range. Despite challenges posed by reduced resolution with depth due to the presence of the skull and fluorescence scattering, advancements such as fluorescent molecular tomography (FMT) combined with computed tomography (CT) have enabled imaging of amyloid deposits in mice without invasive procedures [29]. Although it is unlikely that the entire human brain can ever be imaged by optical methods, it is currently feasible to visualize the cortical surface where most amyloid deposits reside, providing valuable diagnostic information [30,31]. Additionally, alternative imaging sites not shielded by the skull, including the retina and the olfactory epithelium, offer promising avenues for detecting amyloid pathology [32,33,34].

Besides their diagnostic relevance, Aβ proteins have also been shown to be relevant therapeutic targets in AD [35,36]. Despite the significant therapeutic advances made for many diseases over the last decades, Alzheimer’s disease and other neurodegenerative diseases remain incurable and continue to pose substantial challenges to medicinal chemists [37]. Moreover, in spite of large investments in drug discovery against these maladies, this effort has paradoxically led to fewer successful drugs [38]. Since acting selectively on one of the pathological hallmarks, as done by the current drugs, has led to little progress, a multitarget approach presents itself as a more effective alternative [39,40,41]. This is the approach that we have taken here, focusing on the inhibition of both β-amyloid and tau aggregation.

Although numerous fluorescent probes with strong affinity for amyloid plaques have been developed, most are unsuitable for clinical use due to issues such as low sensitivity and specificity, toxicity or inability to cross the blood brain barrier [42]. Several years ago, our group reported styrylquinoline **A**, which showed the ability to inhibit Aβ_42_ aggregation in a ThT-based fluorometric assay, acting as a moderate fibrillization inhibitor and amyloid binder. From a spectroscopic perspective, the native fluorescence of compound **A** is highly dependent on solvent polarity, with its emission in ethanol (mimicking the protein environment) showing a red shift compared to more polar solvents. Upon binding to Aβ_42_, **A** exhibits a hypsochromic shift and increased fluorescence intensity. Additionally, its hydrochloride salt displays a solid-state fluorescence emission maximum above 600 nm [43]. Wang and co-workers reported a series of 2-styrylquinoline derivatives as multifunctional agents for Alzheimer’s disease treatment that inhibit Aβ_1–42_ aggregation, act as antioxidants, and chelate biometals, although their potential as diagnostic agents was not investigated [44]. Styrylquinoline is a privileged scaffold in medicinal chemistry [45,46], and derivatives of the styrylquinoline core have been proved to inhibit several pathological pathways involved in AD. Styrylquinoline derivatives have also identified as imaging probes via SPECT (Single-photon emission computed tomography) to detect Aβ plaques [47].

In this context, we viewed compound **A** as a suitable starting point for manipulation towards the development of small-molecule theranostic agents for protein misfolding diseases. Theranostics, i.e., the combination of diagnostic and therapeutic capabilities in a single agent, is one of the current frontiers in therapeutics, but research in this area is generally restricted to the use of nanoparticle-based radiotracers for the treatment of cancer [48]. Small-molecule theranostics, on the other hand, have received relatively little attention, especially in therapeutic areas different from cancer [49,50,51].

**Figure 1:**
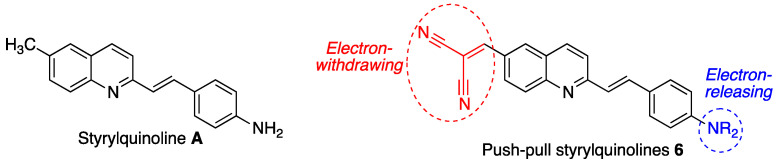
Design of styrylquinolines **6** and their push-pull architecture.

From the point of view of optical imaging purposes, we reasoned that the absorbance and emission maxima in styrylquinolines should be readily altered by incorporating a push-pull architecture, thereby strengthening the molecule’s electron-donating and -accepting properties to amplify the bathochromic shift in its spectrum. To this end, we designed the highly conjugated compounds **6**, bearing electron-releasing dialkylamino groups and electron-withdrawing dicyanomethylene groups at the ends of the molecule (Figure 1).

## 2. Results and Discussion

### 2.1. Synthesis of Compounds ***6***

**Scheme 1:**
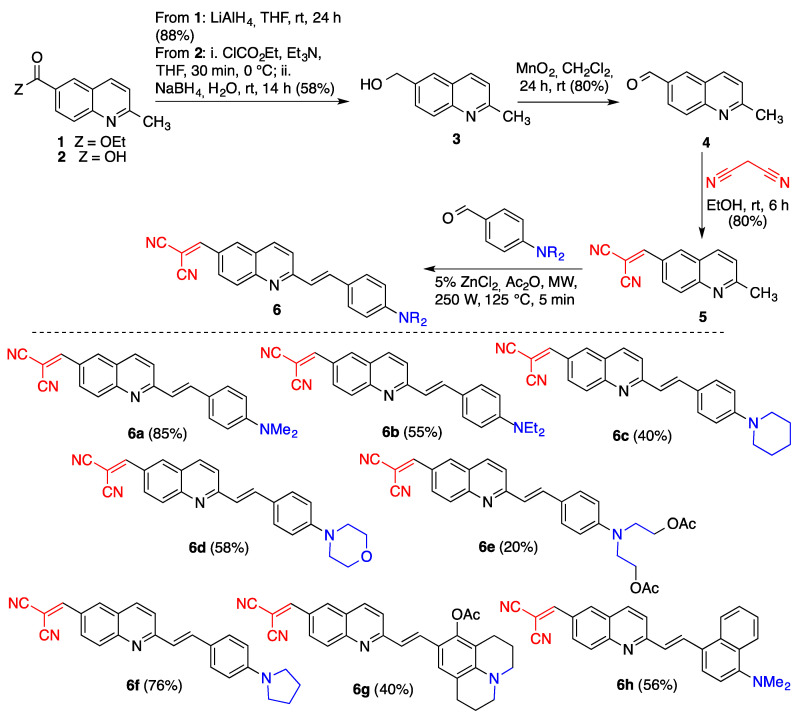
Synthesis of compounds **6**. The electron-releasing and electron-withdrawing components of the push-pull system are shown, respectively, in blue and red.

For the preparation of the target compounds, we employed the four-step route shown in Scheme 1, which, for higher efficiency, was designed to generate structural variability at the last step. The starting materials were the commercially available C-6 functionalized quinolines **1** and **2**. In the case of ester **1**, treatment with lithium aluminum hydride in tetrahydrofuran (THF) gave alcohol **3** in 88% yield. On the other hand, the carboxylic acid derivative **2** needed to be first activated by its transformation into a mixed anhydride by treatment with ethyl chloroformate, followed by in situ NaBH_4_ reduction to give **3** in 58% yield. This alcohol was next oxidized with manganese oxide suspended in dichloromethane to give aldehyde **4** in 80% yield. All attempts to directly transform **1** into **4** by partial reduction with DIBAL were unsuccessful. The Knoevenagel condensation of **4** with malononitrile afforded an 80% yield of compound **5**, which was finally coupled with several aromatic aldehydes bearing a *p*-disubstituted amino group through an aldol condensation promoted by acetic anhydride and zinc chloride as a Lewis acid catalyst [52], to give the desired derivatives **6**. These compounds showed an excellent stability, even upon long-term storage, which allowed their photophysical and pharmacological characterization through the studies described below.

### 2.2. Spectral Properties of Compounds ***6***

#### 2.2.1. Spectrophotometric UV-Vis Absorption Properties and Proton Transfer Reactions

**Figure 2:**
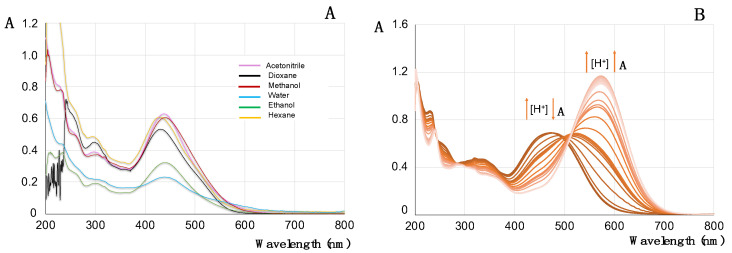
(**A**) UV-Vis absorption spectra of compound **6f** at 1.0 × 10^−5^ M concentration in several solvents (A, absorbance). (**B**) Effect of increasing HCl concentration on the UV-Vis absorption spectra of compound **6f** (5.0 × 10^−5^ M) in ethanol.

Compounds **6** were first characterized by UV-Vis absorption spectrophotometry in ethanol. They all showed three absorption bands (230–250 nm, 320–350 nm and 420–500 nm), which vary slightly according to the nature of the substituents. In order to study the relationship between the spectroscopic properties of compounds **6** and the polarity of their environments, absorption spectra were obtained in solvents with varying polarity. As a representative example, Figure 2A shows the UV-Vis absorption spectra of **6f** in different solvents, showing no significant changes in the position of the absorption maxima. This behavior was observed for all compounds, with the exception of a slight bathochromic shift detected when dissolving compound **6h** in water (Figure S1, ESI). Since they are required in order to adequately monitor compound concentrations, the molar absorptivities of all compounds at each absorption maximum were determined and are summarized in Table S1.

The influence of the protonation of basic nitrogen atoms on the UV-Vis absorption spectra was next studied, and significant spectral changes were observed that allow the use of these compounds as chromogenic sensitive pH sensors. This behavior is shown in Figure 2B, using ethanol as solvent. It can be readily observed that protonation creates a new absorbance band having a maximum around of 580 nm, whose intensity increases with lowered the pH values. On the other hand, the absorption band with a maximum around 430–450 nm decreases upon protonation. Figures S2 and S3 show the influence of the proton transfer reaction on the absorption spectra in dioxane and water respectively. The behavior is similar to the one described in ethanol, although in the case of aqueous solutions, the absorbance values are lower for all the compounds studies and there is not a clear isosbestic point as found in the case of organic solvents, suggesting the existence of more than one species in the proton transfer equilibrium reaction.

#### 2.2.2. Influence of Solvent Polarity and Acid-Base Equilibria on Fluorescence Emission

**Figure 3:**
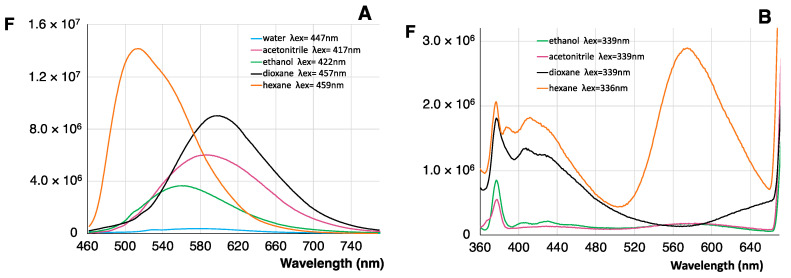
(**A**) Fluorescence emission spectra of compound **6e** (λ_ex_ = 420–450 nm) in solvents with varying polarity. (**B**) A similar study performed for **6h** at λ_ex_ = 339 nm. Concentrations: 2.5 × 10^−7^ M for hexane and dioxane; 1.0 × 10^−6^ M for acetonitrile and ethanol; 5.0 × 10^−6^ M for water. F: Fluorescence in arbitrary units.

Contrary to the behavior of absorption maxima, the position of the fluorescence emission maxima was found to be very sensitive to solvent polarity, a feature that is of great relevance from a sensing point of view, as it ensures that changes in the environment will affect the fluorescence emission in a significant way. Thus, Figure 3A shows the fluorescence emission spectra of compound **6e**, as a representative example, in solvents of varying polarity. It can be observed that the position of the fluorescence emission maximum is shifted from a shorter wavelength in hexane, slightly shifted towards the red in ethanol and acetonitrile and red-shifted in dioxane (600–700 nm). It is relevant to note that the position of the fluorescence maximum in water is close to that observed in ethanol, and that the fluorescence intensity in water is between 10 and 100 times lower than that observed in ethanol or acetonitrile. On the other hand, as exemplified in Figure 3B for compound **6h**, when the excitation wavelength corresponds to the absorption band with a maximum in the 290–350 nm region, two emission bands can be observed, at 350–450 nm and 550–600 nm, with similar intensities in acetonitrile an ethanol but with a significantly higher intensity for the emission at 570 nm in the case of a low polarity solvent such as hexane.

All compounds showed a similar behavior in terms of fluorescent emission, and the spectra corresponding to **6b** and **6c** are shown in Figures S4 and S5. The emission wavelengths for all compounds in different solvents are summarized in Table S2.

The appearance of a fluorescence emission band at longer wavelengths can be attributed to a process of intramolecular charge transfer (ICT) between the donor and acceptor groups. ICT processes appear sometimes in structures with a high degree of π-conjugation and which also possess acceptor and donor groups, showing more than one emission band that is more or less favoured by environmental factors, such as the polarity or viscosity of the medium [53,54] and are of interest because ICT is very sensitive to environment. ICT can be hindered because of lack of resonance/coplanarity between the donor and acceptor groups of the molecule enables the emission of only the main emission band, however in certain environments of higher stiffness or viscosity, such as those provided by hydrophobic and beta-conformational folding of certain proteins, ICT processes can be favored. For this reason, a more detailed study of this process was undertaken for our compounds, due to the protonation of the electron-donating groups involved in the ICT should prevent or decrease the emission corresponding to this process.

**Figure 4:**
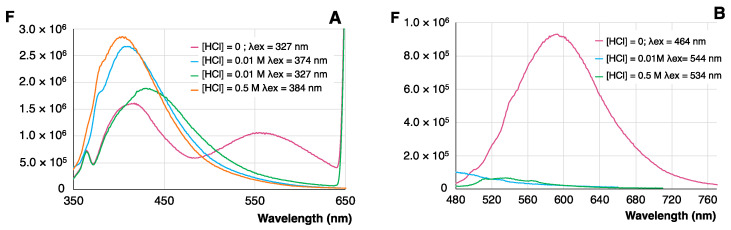
(**A**) Alterations in the fluorescence emission spectra of compound **6a** (λ_ex_ = 330–380 nm) in acetonitrile and in the presence of increasing amounts of HCl. (**B**) The same experiment, performed at λ_ex_ = 460–550 nm. F: Fluorescence in arbitrary units.

In order to demonstrate the existence of an ICT process, the influence of acid addition on the spectral behavior of the styrylquinoline derivatives was studied, since the protonation of the basic nitrogens should prevent the ICT. Thus, when HCl was added, a decrease in the fluorescence intensity of the 550–600 nm band was observed together with an increase in the fluorescence intensity of the base fluorophore band due to the quinoline fragment. This experiment, performed on compound **6a**, is summarized in Figure 4 and Figures S6–S9. The HCl titrations showed a decrease in the intensity of the red-shifted (550–600 nm) emission band, until it disappears, and a progressive increase of the emission band in the region of 400–450. In parallel, the effect of HCl addition was studied by absorption spectrophotometry in the UV-Vis region. Protonation leads to the appearance of new red-shifted absorption bands. The presence of isosbestic and isoemissive points shows that there are only two species involved in the proton transfer equilibria. The acid titrations monitored by spectrofluorimetry are shown in detail in Figures S6–S11.

### 2.3. Sensing of β-Amyloid Protein Fibrils

The process of developing oligomers, fibrils, and aggregates of beta-amyloid protein is highly sensitive to external factors such as ionic strength [55], or the presence of divalent cations [56]. To minimize the effect of cations, chelating agents were used in the preparation of buffer solutions (see Section 3.3). Incubation time is another crucial factor. Therefore, prior to testing each compound **6**, the reproducibility of the formation of beta-amyloid fibrils and aggregates has been verified with thioflavin. In order to explore the fluorescent sensing properties of compounds **6** for the detection of beta-amyloid protein fibrils, the gold standard thioflavin T (ThT) was used as a reference compound. Titration of ThT with beta-amyloid fibrils leads to the appearance of the characteristic ThT fluorescence. Figure S12 shows the verification of the formation of beta-amyloid protein fibrils using ThT as a fluorescent sensor through the increase in fluorescence intensity of ThT when beta-amyloid fibrils are present, either in 1:1 or 2:1 protein:sensor ratios.

**Figure 5:**
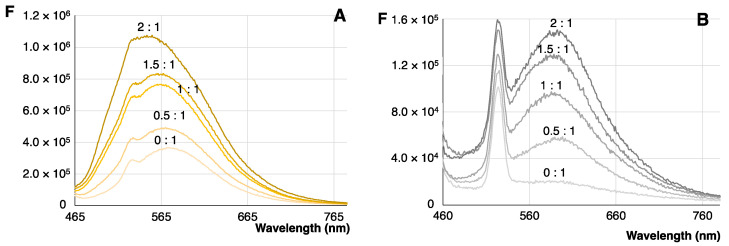
(**A**) Titration of compound **6e** with increasing concentrations of amyloid fibrils of human β-amyloid peptide (fragment 1–42) at the excitation wavelength λ_ex_ = 447 nm. (**B**) Titration of compound **6h** with increasing concentrations of amyloid fibrils of human β-amyloid peptide (fragment 1–42) at the excitation wavelength λ_ex_ = 443 nm. F: Fluorescence in arbitrary units. Sensor concentration 1.0 × 10^−6^ M. The protein:sensor molar ratios are shown over each spectra.

All the styrylquinoline derivatives **6** showed a marked increase in fluorescence intensity in the presence of increasing concentrations of beta-amyloid protein fibrils. This increase was higher when measured under the experimental conditions corresponding to the ICT emission band (560–610 nm depending on the compound), which also corresponds to emission in the vicinity of red. Figure 5A,B show the effect of the presence of increasing concentrations of beta-amyloid fibrils on the fluorescence emission of compounds **6e** and **6h**. Since the value of λ_ex_ was 447 nm (420–450 nm considering the whole library), this result represents a high Stokes shift, with clear advantages from an analytical point of view, also considering the use of fluorescence microscopy for visualizing emission in sample tissues.

**Figure 6:**
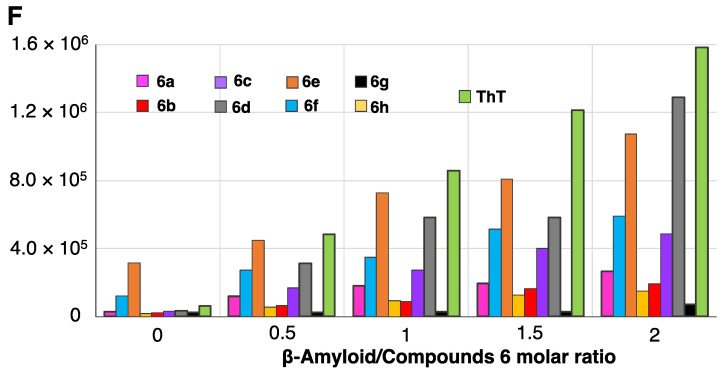
Enhancement of the fluorescence emission of styrylquinoline derivatives **6** following their interaction with amyloid fibrils of human β-amyloid peptide (fragment 1–42) at the excitation wavelength characteristic of each compound. F: Fluorescence in arbitrary units.

A significant enhancement in fluorescence emission was observed for all the styrylquinoline derivatives **6** in the presence of beta-amyloid protein fibrils, with the increases in fluorescence of compounds **6d** and **6e** being in a similar order of magnitude as those of the ThT standard. Both compounds have in common the presence of oxygen atoms in the electron-releasing moiety, which may contribute to a better adaptation to the abnormal beta-folding structure of the protein. On the other hand, compounds **6g** and **6h**, with more extended aromatic systems at the donor moiety, seem to have greater difficulty in binding to the beta sheet structure of the protein. This behavior is summarized in Figure 6.

**Figure 7:**
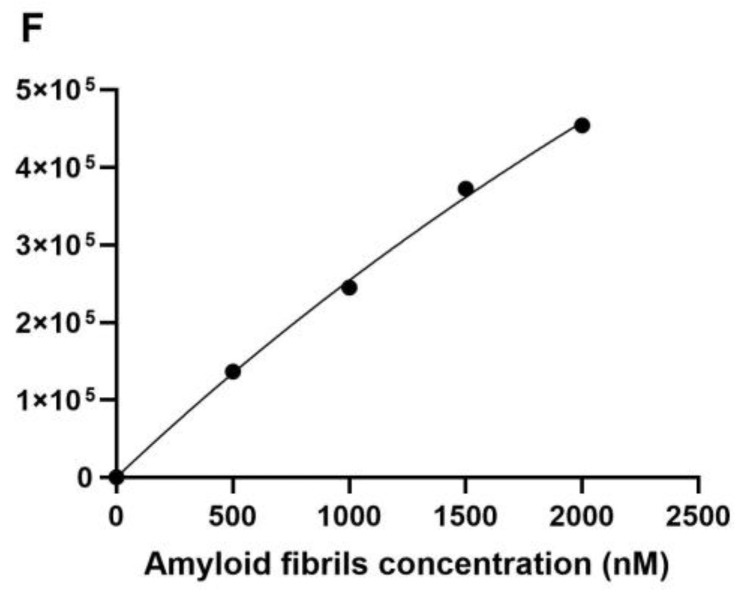
An example of dissociation constant determination, based on the variation (increase) of the fluorescence intensity (F) of compound **6c** in the presence of increasing concentrations of fibrils of β-amyloid peptide; λ_ex_ = 429 nm, λ_em_ = 540 nm.

The binding constants of compounds **6** with β-amyloid fibrils were determined using a non-linear fitting method, as illustrated in Figure 7 for the case of compound **6c**. The *K*_d_ values were obtained at the excitation and emission maxima values corresponding to the ICT band close to or inside the NIR region, which gives a better fit than shorter wavelengths. The quantitative values of binding constants are very sensitive to the protocol used for the formation of amyloidogenic protein aggregates. Thus, several rather divergent *K*_d_ values (μM) have been reported in the literature for the ThT-β-amyloid protein dissociation constant, namely 0.8 [57], 2.56 [58], 30.35 [59] or 46 [60]. Our experimental value of 16.76 (see Table 1) can be considered average. A comparison of the values of the dissociation constants shows a higher affinity for the Aβ peptide for all compounds **6** than the ThT reference. All *K*_d_ values are in the μM range (Figures S13 and S14 and Table 1), showing sufficient affinity to label the protein but also proving the reversibility of the process, an essential feature in a sensor. The compound with the highest affinity for amyloid fibrils is the pyrrolidine derivative **6f**, presumably because its higher rigidity when compared with dialkylamino derivatives, combined with a relatively small molecular size, allows a tighter fitting in the planar space created by β-sheets.

### 2.4. Ex Vivo Staining Experiments on β-Amyloid Plates

To complete the characterization of compounds **6** as β-amyloid probes, we examined the ability of compounds **6b** and **6c** to stain insoluble aggregate plaques in several types of AD tissues, namely brain samples from APP/PS1 (amyloid precursor protein/presenilin 1) transgenic mice and human Alzheimer’s disease patients. These studies were performed using double immunofluorescence techniques coupled to visualization by confocal microscopy.

**Figure 8:**
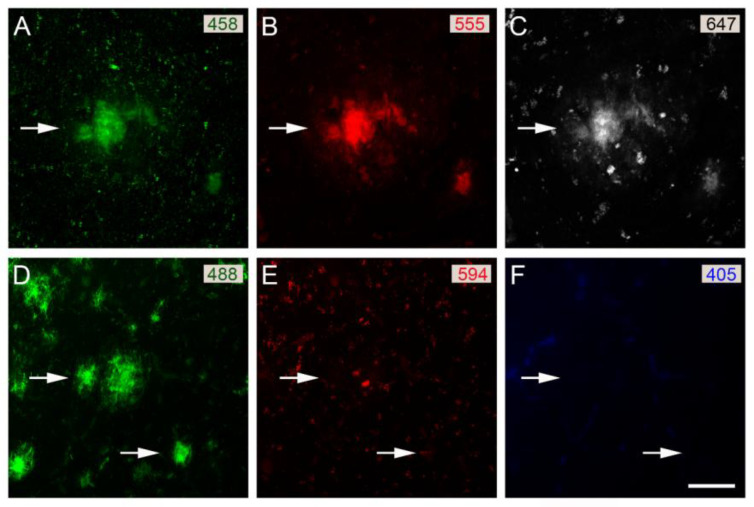
The labeling of amyloid-beta deposits with compound **6b** in the somatosensory cortex of APP/PS1 transgenic mice was evaluated using Z-projection images from two separate confocal stacks (**A**–**F**). These representative images show amyloid-beta deposits labelled with compound **6b**. Positive staining of amyloid-beta plaques was observed at excitation wavelengths of 458 nm (**A**), 555 nm (**B**), 647 nm (**C**), and 488 nm (**D**), whereas no amyloid-beta deposits were detected at 594 nm (**E**) or 405 nm (**F**). White arrows highlight several amyloid-beta deposits, and the scale bar in panel (**F**) represents 20 µm.

Compound **6b** was chosen for an initial labeling study by assessing its binding properties in the somatosensory cortex of APP/PS1 transgenic mice, a model of Alzheimer’s disease that develops amyloid-beta deposits in the brain from 6 to 7 months of age. The results obtained demonstrated specific binding to β-sheet amyloid structures, as evidenced by detection at multiple excitation wavelengths (Figure 8). Amyloid-beta plaques were most distinctly labeled at an excitation wavelength of 458 nm, with effective staining also being observed at 488 nm and 555 nm (Figure 8A–D). In contrast, no staining was detected when exciting at 594 nm or 405 nm (Figure 8E,F).

**Figure 9:**
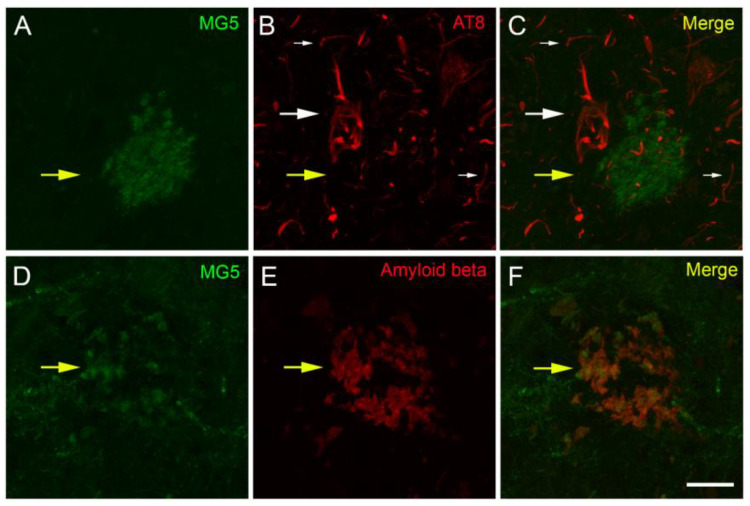
Characterization of compound **6b** labeling in the temporal cortex of human AD patient samples was performed using Z-projection photomicrographs from confocal image stacks. Panels (**A**,**D**) show amyloid-beta deposits labeled with compound **6b**. Labeling with the AT8 antibody, which detects phosphorylated tau, is displayed in panel (**B**), while panel (**E**) shows labeling with an amyloid-beta antibody. Panel (**C**) is a merged image of (**A**,**B**), and (**F**) is a merged image of (**D**,**E**). Small white arrows highlight some phosphorylated tau fibrils, large white arrows mark paired helical filaments and yellow arrows point to the core of the amyloid-beta plaque. The white scale bar in panel (**F**) corresponds to 20 µm.

Building on this preliminary study, we examined compounds **6b** and **6c** as staining agents for aggregates in the temporal neocortex of an Alzheimer’s disease patient. Both compounds specifically labeled amyloid-beta plaques but did not label neurofibrillary tangles. As shown in Figure 9, no colocalization was observed between structures labeled with the AT8 antibody (which detects phosphorylated tau) and compound **6b**. This represents a key difference with Thioflavin S (ThS), which is known to bind both beta-sheet structures and phosphorylated tau in brain samples from AD patients [61]. Furthermore, consistent with the mouse studies, the binding of compound **6b** to amyloid-beta was confirmed by its colocalization with an amyloid-beta antibody (Figure 9D–F). A comparable analysis with compound **6c** also demonstrated its strong binding affinity to the plaque core, as illustrated in Figure S15. The selectivity for amyloid beta deposits over phosphorylated tau represents an advantage for the application of the compounds as sensors over the known molecules, such as ThT, which lack selectivity.

### 2.5. Pharmacological Experimental Protocols

#### 2.5.1. Effect of Compounds **6** on Cell Viability

**Figure 10:**
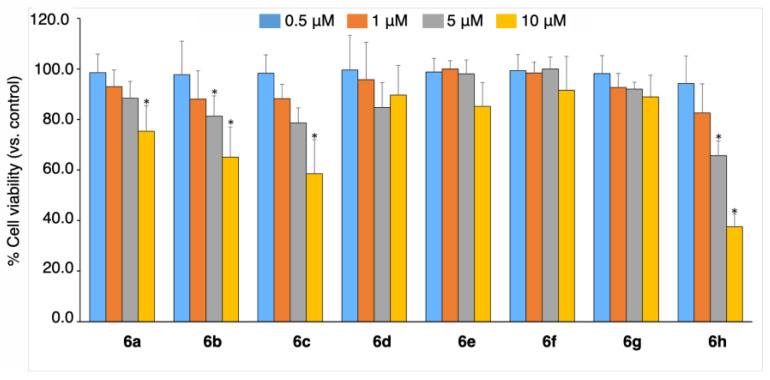
Effects of styrylquinolines on cell viability. Data are expressed in percentage of control HEK293-Tau3R cells and represent the mean ± SEM of three independent experiments performed in triplicates. The symbol * indicates significant differences of styrylquinolines treated cells versus non-treated control cells (*p* < 0.05) (Student–Newman–Keuls test).

The stable HEK/tau cell line, which overexpresses wild-type human tau (4R1N) in HEK293 cells, displays a moderate level of tau phosphorylation. This phosphorylation can be further elevated by treatment with okadaic acid, leading to tau hyperphosphorylation and providing a relevant Alzheimer’s disease model linked to tau aggregation. The cytotoxic effects of compounds **6** were evaluated in HEK293-Tau3R cells, characterized by tau protein overexpression and which were used for subsequent neuroprotection studies, using the MTT assay. Compounds **6** were tested at concentrations ranging from 0.1 to 10 µM, and most exhibited cytotoxicity below 80% at 5 µM (Figure 10). It should be noted that HEK293-Tau3R cells are significantly more susceptible to compound-induced reductions in viability compared to more robust cell lines such as SH-SY5Y [62].

#### 2.5.2. Experimental Protocol for Aβ_1–42_ Aggregation Inhibition

**Figure 11:**
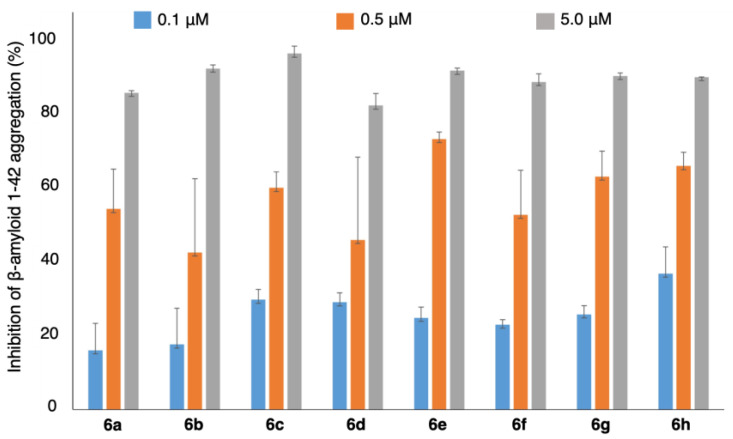
Effects of styrylquinolines **6** on Aβ_1–42_ aggregation. Fibril formation was determined by ThT fluorescence in the absence and presence of compounds **6**. Data are given as the percentage of inhibition of aggregation ± SEM for each styrylquinoline concentration of three separate experiments. All compounds, at all concentrations assayed, significantly inhibited β-amyloid aggregation (*p* < 0.05 versus 2 μM Aβ_1–42_, using the Student–Newman–Keuls test).

After confirming that compounds **6** interact with β-amyloid fibrils, their ability to inhibit Aβ_1–42_ aggregation was demonstrated by measuring the decrease that they cause in the fluorescence intensity of a positive control formed by Aβ_1–42_ fibrils with Thioflavin T (ThT). This effect was observed for all compounds tested at concentrations ranging from 0.1 to 5 μM. At the highest concentration (5 μM), all compounds reduced ThT fluorescence by up to 80%. The choice of ThT over other possible fluorescent dyes, such as Congo red or *trans*-2-[4-(dimethylamino)styryl]-3-ethyl-1,3-benzothiazolium perchlorate (DMASEBT) was based on its better aqueous solubility in comparison with the former and its ready commercial availability as regards the latter [63,64]. As shown in Figure 11, the most active compound at a 0.5 μM concentration, which is non-toxic for all compounds, is **6e**, closely followed by the more rigid julolidine derivative **6g** and the naphthalene derivative **6h**.

Regarding the possibility of assay interferences in the ThT based experiments, the fluorescence of compounds **6** is very weak in aqueous solution, and at a concentration of 5 × 10^−6^ M, no true fluorescence maximum is observed in the 550–600 nm region when exciting at 450 nm (see Figure 3, Figures S4 and S5). In the absence of fibrils, the fluorescence of ThT is also negligible. In the presence of fibrils and exciting at 450 nm (λ_ex_ of ThT and most compounds **6**), the fluorescence intensity of ThT at 500 nm should not be significantly affected, since all compounds have their maximum emission above 550 nm. Under the conditions of the Aβ aggregation inhibition assay, a protein concentration of 2 μM produces a fluorescence intensity for ThT of 16 × 10^6^ (arbitrary units) at 500 nm, and the emission intensities of compounds **6** at a concentration of 1 μM and in the presence of fibrils at that wavelength are between 100 and 200 times lower, so the potential for interference in the inhibition assay is negligible. At 0.1 and 0.5 μM concentrations, the influence of compounds **6** on ThT fluorescence is also negligible. Our compounds, having somewhat lower *K*_d_ values than ThT for Aβ fibrils, could conceivably displace it from its binding to the fibrils, potentially interfering with the experiment. However, this possibility can be ruled out by the fact that the concentration of ThT is 2–100 times higher than that of compounds **6**.

#### 2.5.3. Experimental Protocol for AcPHF6 Aggregation Inhibition

This in vitro assay was performed using the *N*-acetylated and *C*-amidated hexapeptide AcPHF6 (Ac-VQIVYK-NH_2_), derived from the ^306^VQIVYK^311^ sequence found in the native tau-hexapeptide. This region of the tau protein is involved in fibril aggregation processes and is commonly used as a model for screening tau aggregation inhibitors [65].

**Figure 12:**
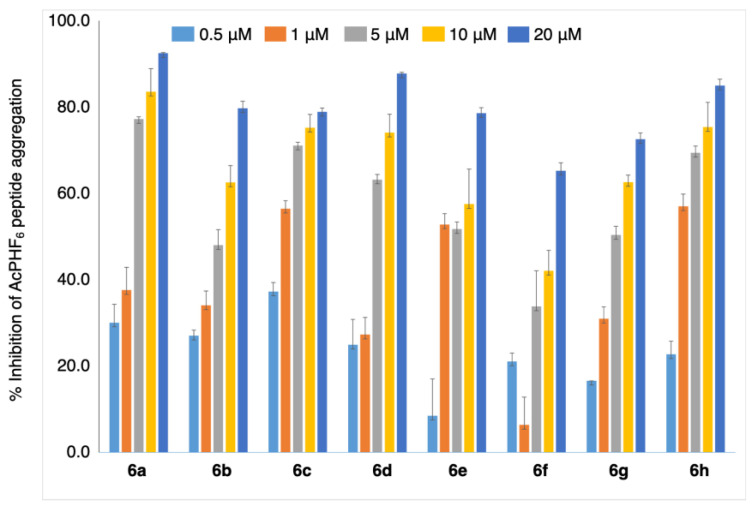
Effects of styrylquinolines **6** on AcPHF6 aggregation. Fibril formation was evaluated by measuring Thioflavin T (ThT) fluorescence in both the absence and presence of styrylquinolines, and the results are reported as the percentage inhibition of aggregation (mean ± SEM) for each styrylquinoline concentration, based on three independent experiments. All compounds, at all concentrations assayed, significantly inhibited tau hexapeptide aggregation (*p* < 0.05 versus 10 μM AcPHF6, using the Student–Newman–Keuls test).

Styrylquinolines **6**, along with methylene blue as a positive control, were tested across a 0.5–20 μM concentration range. A decrease in fluorescence intensity, compared to AcPHF6 alone, indicated that these compounds effectively inhibited tau-hexapeptide aggregation at concentrations as low as 0.5 μM (Figure 12). Again, the best result at a non-toxic concentration (1 μM) was found for compound **6e**, accompanied by the naphthyl derivative **6h** and the piperidine derivative **6c**.

#### 2.5.4. Neuroprotective Effect of Compounds **6** in a Model of Neurotoxicity Induced by Okadaic Acid

**Figure 13:**
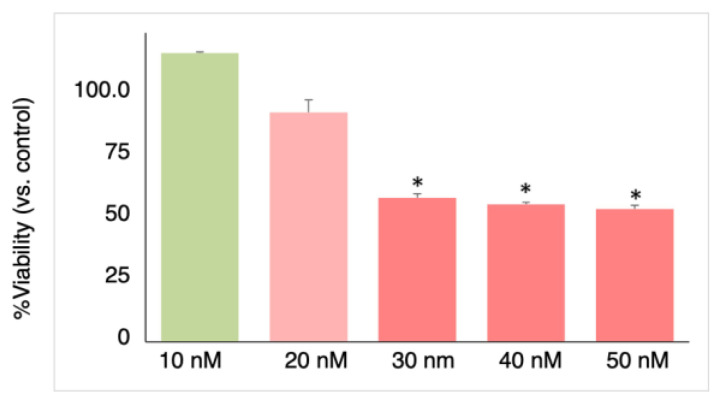
Effects of okadaic acid on cell viability at the concentration range of 10–50 nM. Data are expressed in percentage of control HEK293-Tau3R cells and represent the mean ± SEM of three independent experiments performed in triplicates. The symbol * indicates significant differences of okadaic acid treated cells versus non-treated control cells (*p* < 0.05) (Student–Newman–Keuls test).

To assess the neuroprotective properties of compounds **6**, a neurotoxicity model using okadaic acid as the insult was established. To this end, HEK293-Tau3R cells were first exposed to okadaic acid at concentrations ranging from 10 to 50 nM for 24 h. A marked decline in cell viability was observed at concentrations above 30 nM (Figure 13).

In Alzheimer’s Aβ plaques and hyperphosphorylated tau tangles can trigger apoptotic signaling, including the activation of caspase-3. A compound that binds to Aβ or tau may prevent aggregation or promote disaggregation, thereby reducing upstream apoptotic signaling. This results in reduced caspase-3 activation as a downstream effect, not by direct caspase-3 inhibition but by modulation of the upstream environment, ultimately affecting observed caspase-3 activity in cell-based assays. Since these pathways are intracellular, a high membrane permeability is needed in order to observe them.

**Figure 14:**
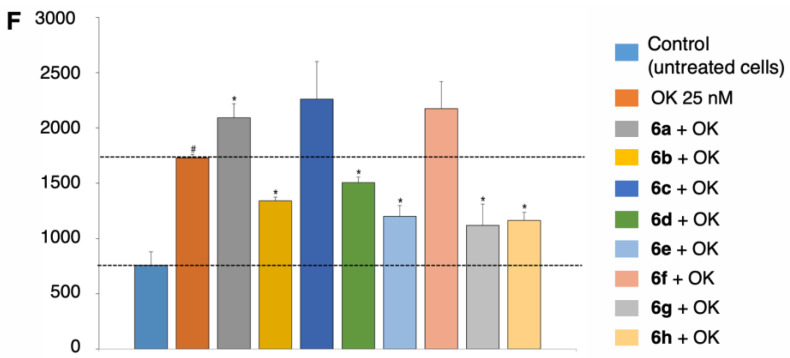
Caspase-3 activity upon 0.1 μM styrylquinoline treatment of okadaic acid (OK) exposed-HEK293-Tau3R cells. Caspase-3 activity was determined by using the fluorogenic substrate Ac-DEVD-AMC. Data are expressed in fluorescence arbitrary units (FAU) and represent the mean ± SEM of three independent experiments. The symbol # indicates significant differences between okadaic acid-treated and untreated HEK293-Tau3R cells (*p* < 0.05). The symbol * indicates significant differences between cells treated with styrylquinolines **6** plus okadaic acid and cells treated with okadaic acid alone (*p* < 0.05) according to the Student–Newman–Keuls test.

In this context, to determine whether cell death was mediated by apoptosis, we measured the levels of caspase 3, which plays a key role in both the intrinsic and extrinsic apoptotic pathways. When cells were treated with 10–50 nM concentrations of okadaic acid, an increase in caspase 3 activity was detected at concentrations up to 25 nM. Given that cell viability decreased up to a concentration of 30 nM okadaic acid, while caspase 3 activity began to rise starting from 25 nM, we selected a 25 nM concentration of okadaic acid to assess the neuroprotective effects of compounds **6** in HEK293-Tau3R cells. Our initial experiments were aimed at confirming that compounds **6** did not alter caspase 3 activity under normal conditions. Subsequently, cells were co-treated with 25 nM okadaic acid and compounds **6** at either 10 or 100 nM concentrations, and it was observed that **6b**, **6d**, **6e**, **6g**, and **6h** significantly reduced caspase-3 activation induced by okadaic acid in HEK293-Tau3R cells. For example, treatment with a 100 nM concentration of compound **6g** decreased caspase-3 activity by more than 50% compared to cells treated with okadaic acid alone (*p* < 0.05), with very similar results being found for **6e** and **6h** (Figure 14).

Considering all the pharmacological data in the aggregate, compound **6e** seems the one showing the most promising profile as a candidate for further development. Compound **6h**, although also interesting, displays a higher cytotoxicity.

## 3. Materials and Methods

### 3.1. General Experimental Information

NMR spectra were recorded on a Bruker Avance 250 spectrometer operating at 250 MHz for ^1^H and 63 MHz for ^13^C, using standard Bruker software (Unidad de Resonancia Magnética Nuclear, Universidad Complutense). Combustion elemental analyses were performed by the Unidad de Microanálisis Elemental at Universidad Complutense, using a Leco-932 instrument. Infrared spectra were obtained with an Agilent Cary630 FTIR spectrophotometer equipped with a diamond accessory for both solid and liquid samples. Microwave-assisted reactions were conducted in a CEM Discover SP microwave reactor. Reaction progress was monitored by thin layer chromatography on aluminum plates coated with silica gel and having a fluorescent indicator (Macherey-Nagel Xtra SIL G/UV254). Flash chromatographic separations were carried out using silica gel (Scharlau 40–60 μm, 230–400 mesh ASTM) or neutral alumina (Merck S22). Melting points were measured on a SMP3 Stuart Scientific instrument and are reported without correction. UV-Vis spectra were obtained with a Cary 60 UV–Vis spectrophotometer from Agilent (Santa Clara, CA, USA), equipped with control and data acquisition Cary WinUV 5.1 software. Uncorrected and corrected excitation and emission spectra, along with measurements at fixed wavelengths, were recorded with a Horiba-Jobin Yvon FluoroMax-4 spectrofluorometer (Kyoto, Japan) equipped with the FluorEssence 2.1 control and data acquisition software. Excitation and emission slits were set at 5 nm. For all experiments, quartz cells with 1 cm of path length were used. Protein samples were incubated using an Accublock D-1301 digital dry bath from Labnet (Madrid, Spain).

Reagents: Synthetic reagents were purchased from Merck-Sigma-Aldrich. Solvents were purchased from Scharlau and Fischer. Thioflavine and amyloid β protein fragment 1–42 (M_W_ = 4514.04) were purchased from Merck-Sigma-Aldrich. The synthetic AcPHF6 peptide (Ac-VQIVYK-NH_2_) was purchased from Bachem (Bubendorf BL, Switzerland). Analytical- or spectroscopic-grade solvents were purchased from SDS and Panreac and were used without further purification. Ultrapure water was obtained using a Milli-Q Direct 8 system (Millipore).

### 3.2. Synthesis

#### 3.2.1. (2-Methylquinolin-6-yl)methanol (**3**)

Method A: Lithium aluminium hydride (10 eq) was suspended in dry THF and the suspension was cooled down to 0 °C using an ice-water bath. A solution of ethyl 2-methylquinoline-6-carboxylate (2 g, 9.29 mmol) in dry THF (20 mL) was slowly added, under argon, to the cooled suspension. After stirring at room temperature for 24 h, the mixture was quenched by gradual addition of ethyl acetate (30 mL), followed by water (2 mL). The resulting suspension was filtered, and the filtrate was extracted with ethyl acetate, which was dried with anhydrous Na_2_SO_4_ and evaporated *in vacuo* to yield compound **3**, which was used in the next step without further purification. Yield: 1.414 g (88%), as a yellowish solid.

Method B: A solution of 2-methylquinoline-6-carboxylic acid (1.5 g, 8 mmol), acetyl chloride (1.2 eq) and triethylamine (1.2 eq) in THF (45 mL) was prepared and stirred at 0 °C for 30 min. After completion of the formation of the mixed anhydride intermediate, NaBH_4_ (1.210 g, 32 mmol) and water (10 mL) were added and the reaction mixture was stirred overnight at room temperature. The mixture was quenched by stirring for 1 h with 1 M aqueous HCl, and then neutralized with NaHCO_3_ and extracted with ethyl acetate, which was dried with anhydrous Na_2_SO_4_ and evaporated *in vacuo* to furnish compound **3**. Yield: 0.802 g (58%).

Characterization data: Mp 118–121 °C. IR (neat) 3172, 1061, 823 cm^−1^. ^1^H NMR (250 MHz, CDCl_3_) δ 2.76 (s, 3H), 4.90 (s, 2H), 7.31 (d, *J* = 8.4 Hz, 1H), 7.67 (dd, *J* = 8.4 and 1.2 Hz, 1H), 7.77 (d, *J* = 1.2 Hz, 1H), 8.01 (d, *J* = 8.4 Hz, 1H), 8.05 (d, *J* = 8.4 Hz, 1H). ^13^C NMR (63 MHz, CDCl_3_) δ 25.6, 65.3, 122.7, 125.3, 126.74, 129.1, 129.2, 136.7, 138.8, 147.7, 159.3. Elemental analysis for C_11_H_11_NO (%). Calculated: C 76.28, H 6.40, N 8.09; found: C 76.45, H 6.35, N 7.97.

#### 3.2.2. 2-Methylquinoline-6-carbaldehyde (**4**)

To a solution of alcohol **3** (1.6 g, 7.43 mmol) in dichloromethane (40 mL) was added manganese dioxide (5 eq). The suspension was stirred at room temperature for 24 h and filtered through a pad of Celite. The filtrate was evaporated and the residue was purified by silica gel flash column chromatography, eluting with hexane: ethyl acetate (2:1, *v*/*v*), to provide compound **4**. Yield: 1.27 g (80%), as a yellow solid. Mp 103–106 °C. IR (neat) 1684 (C=O), 823 cm^−1^. ^1^H NMR (250 MHz, CDCl_3_) δ 2.58 (s, 3H), 7.19 (d, *J* = 8.4 Hz, 1H), 7.88 (dd, *J* = 8.4 and 0.5 Hz, 1H), 7.93–8.00 (m, 2H), 8.08 (d, *J* = 1.6 Hz, 1H), 9.95 (d, *J* = 0.5 Hz, 1H). ^13^C NMR (63 MHz, CDCl_3_): δ 26.1, 123.6, 126.3, 127.3, 130.3, 133.8, 134.0, 137.8, 151.0, 162.9, 192.0. Elemental analysis for C_11_H_9_NO (%). Calculated: C 77.17, H 5.30,N 8.18; found: C 77.42, H 5.42, N 8.03.

#### 3.2.3. 2-((2 Methylquinolin-6-yl)methylene)malononitrile (**5**)

To a solution of aldehyde **4** (1.2 g, 4.6 mmol) in ethanol (6 mL) was added malononitrile (0.31, 4.6 mmol). The solution was stirred at room temperature for 6 h and the solvent was evaporated. After washing the residue with 10:1 hexane/ethyl ether, compound **5** was purified by recrystallization from acetone: methanol (5:1). Yield, 0.94 g (80%), as a dark red solid. Mp 190–193 °C. IR (neat) 2222, 1575, 833 cm^−1^. ^1^H NMR (250 MHz, d_6_-DMSO) δ 2.72 (s, 3H), 7.58 (d, *J* = 8.5 Hz, 1H), 8.11 (d, *J* = 9.0 Hz, 1H), 8.28 (dd, *J* = 9.0 and 1.7 Hz, 1H), 8.43 (d, *J* = 8.5 Hz, 1H), 8.49 (d, *J* = 1.7 Hz, 1H), 8.72 (s, 1H). ^13^C NMR (63 MHz, d_6_-DMSO): δ 25.56, 82.0, 113.7, 114.7, 124.1, 126.1, 128.3, 128.9, 130.0, 134.3, 137.9, 149.5, 161.1, 163.2. Elemental analysis for C_14_H_9_N_3_ (%). Calculated: C 76.70, H 4.14, N 19.17; found: C 76.89, H 4.19, N 18.86.

#### 3.2.4. General Method for the Synthesis of Styrylquinolines **6**

A suspension of compound **5** (0.200 g, 0.9 mmol), the suitable aldehyde (1.5 eq) and zinc chloride (0.006 g, 0.045 mmol) in acetic anhydride (1.5 mL) was placed in a pressure-tight microwave tube equipped with a stirring bar. The reaction mixture was microwave-irradiated for 5 min at 125 °C and 250 W. The solvent was evaporated *in vacuo* and the dark purple residue was purified by silica gel flash chromatography, eluting with hexane: ethyl acetate (9:1, *v*/*v*).

(*E*)-2-((2-(4-(Dimethylamino)styryl)quinolin-6-yl)methylene)malononitrile (**6a**). Prepared from 4-(dimethylamino)benzaldehyde (0.200 g, 1.35 mmol). Yield: 0.267 g (85%), as a dark red solid. Mp 188–191 °C. IR (neat) 2224, 1568, 1521, 1164, 818 cm^−1^. ^1^H NMR (250 MHz, CDCl_3_): δ 3.08 (s, 6H), 6.76 (d, *J* = 8.6 Hz, 2H), 7.18 (d, *J* = 16.1 Hz, 1H), 7.58 (d, *J* = 8.4 Hz, 2H), 7.73 (m, 2H), 7.87 (s, 1H), 8.03–8.33 (m, 4H). ^13^C NMR (63 MHz, CDCl_3_): δ 40.6 (2C), 82.1, 112.5 (2C), 113.4, 114.5, 121.4, 123.4, 124.3, 126.9, 128.2, 129.4, 129.7 (2C), 130.8, 133.6, 137.5, 138.3, 151.3, 151.6, 159.3, 160.8. Elemental analysis for C_23_H_18_N_4_ (%). Calculated: C 78.83, H 5.18, N 15.99; found: C 78.69, H 5.28, N 16.16.

(*E*)-2-((2-(4-(Diethylamino)styryl)quinolin-6-yl)methylene)malononitrile (**6b**). Prepared from 4-(diethylamino)benzaldehyde (0.239 g, 1.35 mmol). Yield: 0.176 g (55%), as a dark red solid. Mp 197–200 °C. IR (neat) 2221, 1564, 1518, 1177, 819 cm^−1^. ^1^H NMR (250 MHz, CDCl_3_): δ 1.25 (t, *J* = 6.9 Hz, 6H), 3.46 (d, *J* = 6.9 Hz, 4H), 6.72 (d, *J* = 8.2 Hz, 2H), 7.19 (d, *J* = 16.4 Hz, 1H), 7.57 (d, *J* = 8.2 Hz, 2H), 7.70–7.81 (m, 2H), 7.88 (s, 1H), 8.33–8.10 (m, 4H). ^13^C NMR (63 MHz, CDCl_3_): δ 13.1 (2C), 44.9 (2C), 81.8, 111.8 (2C), 113.4, 114.5, 121.3, 122.8, 123.3, 126.8, 128.1, 129.4, 130.0 (2C), 130.7, 133.6, 137.3, 138.3, 149.2, 151.3, 159.2, 160.9. Elemental analysis for C_25_H_22_N_4_ (%). Calculated: C 79.34, H 5.86, N 14.80; found: C 79.71, H 6.02, N 14.91.

(*E*)-2-((2-(4-(Piperidin-1-yl)styryl)quinolin-6-yl)methylene)malononitrile (**6c**). Prepared from 4-(piperidin-1-yl)benzaldehyde (0.255 g, 1.35 mmol). Yield: 0.126 g (40%), as a dark red solid. Mp 180–183 °C. IR (neat) 2223, 1565,1511, 1172, 1120, 822 cm^−1^. ^1^H NMR (250 MHz, CDCl_3_): δ 1.78–1.64 (m, 6H), 3.28–3.39 (m, 4H), 6.96 (d, *J* = 8.7 Hz, 2H), 7.22 (d, *J* = 16.1 Hz, 1H), 7.57 (d, *J* = 8.7 Hz, 2H), 7.70–7.80 (m, 2H), 7.88 (s, 1H), 8.10–8.30 (m, 4H). ^13^C NMR (63 MHz, CDCl_3_): δ 24.7, 25.9 (2C), 49.7 (2C), 82.1, 113.4, 114.5, 115.6 (2C), 121.4, 124.2, 126.1, 126.9, 128.3, 129.4, 129.5 (2C), 130.8, 133.6, 137.5, 137.9, 151.1, 152.9, 159.2, 160.5. Elemental analysis for C_26_H_22_N_4_ (%). Calculated: C 79.97, H 5.68, N 14.35; found: C 77.43, H 5.66, N 14.82.

(*E*)-2-((2-(4-Morpholinostyryl)quinolin-6-yl)methylene)malononitrile (**6d**). Prepared from 4-morpholinobenzaldehyde (0.257 g, 1.35 mmol). Yield: 0.129 g (35%), as a dark red solid. Mp 179–182 °C. IR (neat) 2227, 1572, 924, 813 cm^−1^. ^1^H NMR (250 MHz, CDCl_3_): δ 3.14–3.25 (m, 4H), 3.75–3.86 (m, 4H), 6.86 (d, *J* = 8.7 Hz, 2H), 7.15 (d, *J* = 16.2 Hz, 1H), 7.51 (d, *J* = 8.7 Hz, 2H), 7.59–7.71 (m, 2H), 7.79 (s, 1H), 7.98–8.20 (m, 4H). ^13^C NMR (63 MHz, CDCl_3_): δ 48.7 (2C), 67.1 (2C), 82.5, 113.3, 114.4, 115.4 (2C), 121.5, 125.2, 127.0, 127.5, 128.4, 129.4 (3C), 131.0, 133.5, 137.5, 137.7, 151.1, 152.3, 159.2, 160.4. Elemental analysis for C_25_H_20_N_4_O (%). Calculated: C 76.51, H 5.14, N 14.28; found: C 76.25, H 5.32, N 14.45.

(*E*)-((4-(2-(6-(2,2-Dicyanovinyl)quinolin-2-yl)vinyl)phenyl)azanediyl)bis(ethane-2,1-diyl) diacetate (**6e**). Prepared from ((4-formylphenyl)azanediyl) bis(ethane-2,1-diyl) diacetate (0.395 g, 1.35 mmol). Yield: 0.070 g (20%), as a dark red solid. Mp 98–100 °C. IR (neat) 2224, 1728, 1516, 1220 cm^−1^. ^1^H NMR (250 MHz, CDCl_3_): δ 2.09 (s, 6H), 3.72 (t, *J* = 6.1 Hz, 4H), 4.30 (t, *J* = 6.1 Hz, 4H), 6.81 (d, *J* = 8.8 Hz, 2H), 7.16 (d, *J* = 16.2 Hz, 1H), 7.56 (d, *J* = 8.8 Hz, 2H), 7.67 (d, *J* = 8.9 Hz, 1H), 7.72 (d, *J* = 16.2 Hz, 1H), 7.85 (s, 1H), 8.08 (d, *J* = 8.9 Hz, 1H), 8.14 (d, *J* = 8.9 Hz, 1H), 8.20 (dd, *J* = 8.9, 1.5 Hz, 1H), 8.27 (d, *J* = 1.5 Hz, 1H). ^13^C NMR (63 MHz, CDCl_3_): δ 21.3 (2C), 50.0 (2C), 61.6, 82.2, 112.4 (2C), 113.4, 114.5, 121.4, 124.0, 125.1, 126.9, 128.3, 129.4 (2C), 129.9, 130.8, 133.6, 137.6, 137.7, 148.7, 151.1, 159.2, 160.5, 171.4 (2C). Elemental analysis for C_29_H_26_N_4_O_4_ (%). Calculated: C 70.43, H 5.30, N 11.33; found: C 70.37, H 4.99, N 11.31.

(*E*)-9-(2-(6-(2,2-Dicyanovinyl)quinolin-2-yl)vinyl)-2,3,6,7-tetrahydro-1*H*,5*H*-pyrido[3,2,1-*ij*]quinolin-8-yl acetate (**6f**). Prepared from 8-hydroxy-2,3,6,7-tetrahydro-1*H*,5*H*-pyrido[3,2,1-*ij*]quinoline-9-carbaldehyde (0.350 g, 1.35 mmol). Yield: 0.156 g (40%), as a dark red solid. Mp 144–146 °C. IR (neat) 2922, 2217, 1750, 1563, 1186 cm^−1^. ^1^H NMR (250 MHz, CDCl_3_): δ 1.83–1.99 (m, 4H), 2.38 (s, 3H), 2.48–2.56 (m, 2H), 2.68–2.70 (m, 2H), 3.12–3.18 (m, 4H), 7.01 (d, *J* = 16.1 Hz, 1H), 7.23 (s, 1H), 7.50 (d, *J* = 8.9 Hz, 1H), 7.61 (d, *J* = 16.1 Hz, 1H), 7.77 (s, 1H), 7.96 (d, *J* = 9.0 Hz, 1H), 8.00 (d, *J* = 8.9 Hz, 1H), 8.10 (dd, *J* = 9.0, 1.9 Hz, 1H), 8.17 (d, *J* = 1.9 Hz, 1H). ^13^C NMR (63 MHz, CDCl_3_): δ 21.1, 21.3, 22.0, 28.0, 30.1, 49.7, 50.3, 81.9, 113.4, 113.5, 114.5, 115.6, 119.9, 121.4, 124.2, 125.3, 126.9, 128.1, 129.3, 130.8, 131.6, 133.6, 137.3, 145.5, 146.9, 151.3, 159.2, 160.6, 169.6. Elemental analysis for C_29_H_24_N_4_O_2_ (%). Calculated: C 75.63, H 5.25, N 12.17; found: C 75.22, H 5.20, N 11.97.

(*E*)-2-((2-(2-(4-(Dimethylamino)naphthalen-1-yl)vinyl)quinolin-6-yl)-methylene)malononitrile (**6g**). Prepared from 4-(dimethylamino)-1-naphthalenecarbaldehyde (0.269 g, 1.35 mmol). Yield: 0.201 g (56%), as a dark red solid. Mp 95–97 °C. IR (neat) 2930, 2105, 1568 cm^−1^. ^1^H NMR (250 MHz, CDCl_3_): δ 3,07 (s, 6H), 7.13 (d, *J* = 8.0 Hz, 1H), 7.37 (d, *J* = 15.9 Hz, 1H), 7.51–7.65 (m, 2H), 7.78 (d, *J* = 8.6 Hz, 1H), 7.83 (s, 1H), 7.89 (d, *J* = 8.6 Hz, 1H), 8.12–8.28 (m, 4H), 8.30–8.35 (m, 2H), 8.63 (d, *J* = 15.9 Hz, 1H). ^13^C NMR (63 MHz, CDCl_3_): δ 45.5 (2C), 82.6, 113.3, 114.1, 114.4, 121.8, 124.3, 125.3, 125.5, 125.7, 126.9, 127.1, 127.8, 128.6, 128.8, 128.9, 129.4, 131.1, 133.2, 133.6, 134.4, 137.8, 151.1, 153.0, 159.2, 160.1. Elemental analysis for C_27_H_20_N_4_ (%). Calculated: C 80.98, H 5.03, N 13.99; found: C 81.17, H 5.32, N 13.50.

(*E*)-2-((2-(4-(Pyrrolidin-1-yl)styryl)quinolin-6-yl)methylene)malononitrile (**6h**). Prepared from 4-(pyrrolidin-1-yl)benzaldehyde (0.236 g, 1.35 mmol). Yield: 0.259 g (76%), as a dark red solid. Mp 123–125 °C. IR (neat) 2851, 2222, 1694, 1568, 1520 cm^−1^. ^1^H NMR (250 MHz, CDCl_3_): δ 2.05 (t, *J* = 6.5 Hz, 4H), 3.38 (t, *J* = 6.5 Hz, 4H), 6.59 (d, *J* = 8.8 Hz, 2H), 7.15 (d, *J* = 16.1 Hz, 1H), 7.55 (d, *J* = 8.8 Hz, 2H), 7.68 (d, *J* = 8.7 Hz, 1H), 7.76 (d, *J* = 16.1 Hz, 1H), 7.85 (s, 1H), 8.07 (d, *J* = 9.1 Hz, 1H), 8.11 (d, *J* = 9.1 Hz, 1H), 8.19 (dd, *J* = 8.9, 1.7 Hz, 1H), 8.27 (d, *J* = 1.7 Hz, 1H). ^13^C NMR (63 MHz, CDCl_3_): δ 25.9 (2C), 48.0 (2C), 81.9, 112.2 (2C), 113.4, 113.6, 121.5, 122.7, 123.6, 126.9, 128.1, 129.4, 129.9 (2C), 130.7, 133.5, 137.4, 138.8, 149.2, 151.4, 159.2, 160.9. Elemental analysis for C_25_H_20_N_4_ (%). Calculated: C, 79.76; H, 5.35; N, 14.88; found: C 79.52, H 5.21, N 14.57.

### 3.3. Preparation of Amyloid β Fibrils from Human Aβ_1–42_ Peptide

Amyloid fibrils from human Aβ_1–42_ peptide were prepared using a procedure that combines several protocols described in the literature [16,59,66]. A 2.2 × 10^−4^ M solution of commercially available Aβ_1–42_ peptide was prepared in a small volume of 100 mM sodium phosphate buffer (pH = 7.04) containing EDTA in concentration 10 mM. This solution was incubated at 37 °C during 72 h in a dry bath supported on an orbital shaker working at 200 rpm. After this time, the necessary volume of 5 mM phosphate buffer (pH = 7.04) was added to achieve a Aβ_1–42_ peptide concentration of 1.0 × 10^−4^ M. The formation of peptide fibrils was then checked by titration of a ThT solution, as described in Section 3.4.3.

### 3.4. Spectrophotometric and Spectrofluorimetric Study of Sensors

#### 3.4.1. General Procedure

The compounds were characterized spectrophotometrically in order to establish the precise sensor/protein molar ratio. A measured amount of each compound was weighed and dissolved in a suitable volume of ethanol to prepare 5.0 × 10^−4^ M solutions. Then, ethanolic solutions of varying concentration in the range of 1.0 × 10^−6^ M to 1.0 × 10^−4^ M were prepared and UV-Vis absorption spectra were obtained. With the data thus obtained, the molar absortivities of each compound were determined.

For the spectrofluorimetric characterization of compounds **6**, 1.0 × 10^−6^ M solutions were prepared from appropriate aliquots of the ethanolic 1.0 × 10^−4^ M stock solution. Then, their excitation and fluorescence emission spectra were obtained.

#### 3.4.2. Influence of Solvent Polarity and pH on Native Fluorescence of Sensors

For the study of the solvatochromic effect, a series of aliquots of the stock solution in ethanol were taken and evaporated at room temperature under reduced pressure and then redissolved in an appropriate volume of the solvents studied: water, acetonitrile, ethanol, dioxane and hexane. Due to the solubility problems of the styrylquinoline derivatives in hexane and water, it was decided to take an aliquot of the ethanolic stock solution and dilute it with the solvent to be studied, so that the proportion of ethanol in the final solution would be 1% or less. The concentration of each compound was adjusted according to the solvent. 2.5 × 10^−7^ M for hexane and dioxane, 1.0 × 10^−6^ M for ethanol and acetonitrile and 5.0 × 10^−6^ M for water. Then fluorescence excitation and emission spectra were recorded.

The effect of acid-base equilibrium on the spectrophotometric and spectrofluorimetric properties of the styrylquinoline derivatives was studied. For this purpose, additions of 10 or 20 μL of HCl were made. The styrylquinoline concentrations were 1.0–5.0 × 10^−5^ M depending on each compound and the solvent studied for the spectrophotometric measurements and 1.0–5.0 × 10^−6^ M spectrofluorimetric measurements. Different concentrations of HCl acid (0.01 M, 0.1 M, 1.0 M and 5.0 M) were tested in order to establish under which experimental conditions protonation is achieved. After each addition, UV-Vis absorption or fluorescence emission spectra were recorded, as appropriate.

#### 3.4.3. Interaction of Compounds **6** with β-Amyloid Fibrils

Solutions (50 μM) of compounds **6**, as well as thioflavin T (ThT), were prepared in phosphate buffer (pH = 7.02) and, after determining their precise concentrations by UV-Vis absorption spectrophotometry using their experimentally determined molar absorptivities, they were adjusted to 1.0 × 10^−6^ M. In the case of ThT, a value of ε = 36,000 M^−1^cm^−1^ (λ_max_ = 412 nm) was used [16]. Subsequently, 10 μL of the freshly prepared amyloid beta fibrils (c = 1.0 × 10^−4^ M) were added to each of the solutions containing the sensors. After a stabilization period of 10 min after each fibril addition at 37 °C, and then equilibration time of 5 min at room temperature, fluorescence emission spectra were recorded for each sensor at their excitation maxima.

The increase in fluorescence intensity shown by compounds **6** when titrating with beta-amyloid fibrils was used to determine the protein/sensor binding constants. Concentration of sensor was fixed at 1.0 × 10^−6^ M and protein concentration varies from 0 to 2.0 × 10^−6^ M. The *K*_d_ values were calculated from the experimental data (obtained in duplicate sets) with GraphPad (GraphPad Software Inc., La Jolla, CA, USA) and the following equation [57,66], where I_F_ are the increase in fluorescence intensity values with respect to the sensor alone and B_max_ is the fluorescence intensity in the plateau region:

$$I_{F}=\;\frac{B_{max}\cdot [\beta -amyloid]}{K_{d}+[\beta -amyloid]}$$

### 3.5. Ex-Vivo Staining Experiments

AD human tissue samples were analyzed within the context of a previous study that utilized fixed coronal sections to investigate the progression of Alzheimer’s disease [67]. Brain tissue samples from AD patients were obtained from the Banco de Tejidos Fundación CIEN (Dr. A. Rábano, Área de Neuropatología, Centro Alzheimer, Fundación Reina Sofía, Madrid, Spain). Immediately after removal, the brains were fixed in cold 4% paraformaldehyde in 0.1 M phosphate buffer (pH 7.4). After 2 h, the tissue was sectioned into small blocks and post-fixed in the same medium at 4 °C for 24–48 h. Serial coronal sections (50 μm thick) were prepared using a vibratome (St. Louis, MO, USA), cryoprotected in a 30% sucrose solution in 0.1 M phosphate buffer and stored in an ethylene glycol/glycerol solution at −20 °C until use. The case used in the present work (VK16; female, 88 years old) was diagnosed with AD based on clinical and neuropathological criteria and classified as Braak stage IV and CERAD stage C.

The free-floating sections were rinsed in 0.1 M phosphate buffer (PB) and incubated for 2 h at room temperature with a 20 μM solution of each compound **6**, prepared by diluting a 1.0 × 10^−4^ M stock solution in DMSO with phosphate buffer. After incubation, the sections were rinsed again in 0.1 M phosphate buffer, mounted using ProLong Gold Antifade Reagent (Invitrogen Corporation, Carlsbad, CA, USA), and stored at −20 °C.

Selected fixed sections were rinsed in 0.1 M PB, pretreated in 2% H_2_O_2_ for 30 min, and incubated for 1 h at room temperature in of 3% bovine serum albumin solution. Subsequently, sections were incubated at 4 °C for 48 h in the same solution with mouse IgG1-anti-phospho-tau, clone AT8 (1:2000, MN1020, Thermo Scientific) or mouse antihuman amyloid beta antibody (1:50, clone 6F/3D; Dako, Glostrup, Denmark). Afterwards, sections were rinsed in 0.1 M PB and incubated at room temperature for 2 h in different solutions containing a goat anti-mouse antibody coupled to Alexa Fluor 594 (1:1000; Molecular Probes, Eugene, OR, USA). Finally, sections were mounted with ProLong Gold Antifade Reagent (Invitrogen Corp).

Image acquisition was performed with a Zeiss LSM 710 confocal laser scanning system (Carl Zeiss Microscopy GmbH, Jena, Germany). They were recorded at 0.5 μm intervals (63 × objective) through separate channels, and ZEN 2012 software (Zeiss) was then used to construct composite images from each optical series by combining the images recorded through the different channels (image resolution 1024 × 1024 pixels; pixel size 188 nm). Z depth in every confocal stack was 40 μm. We scanned 6 image stacks from the CA1 region.

### 3.6. Pharmacological Characterization

#### 3.6.1. Stock Preparation of Aβ_1–42_ Peptide

A 0.5 mg/mL solution of Aβ_1–42_ peptide in 10% aqueous ammonium hydroxide was sonicated for 30 min to achieve monomerization. Solvent evaporation in a SpeedVac afforded a thin film that was dissolved in 60 mM NaOH, adjusting its concentration to 20 μM, as a stock solution. For aggregation inhibition experiments, 2 μM Aβ_1–42_ peptide was mixed with the styrylquinolines **6** and incubated for 4 days at 37 °C. The degree of Aβ aggregation was determined using Thioflavin-T fluorescence analysis.

#### 3.6.2. Aβ_1–42_ Aggregation Inhibition

Briefly, the screen of the styrylquinolines **6** was performed based on Thioflavin T fluorescence. All assays were done on flat black 96-well plates. For the inhibition of aggregation assays, 2 µM peptide Aβ_1–42_ and 10 µM ThT were incubated in in 50 mM phosphate buffer (pH 7.4) containing various concentrations of styrylquinolines (0.1, 0.5 and 5 µM). Peptide Aβ_1–42_ alone in buffer reaction served as a control for the screen. Fluorescence values were measured at 37 °C in replicates using a microplate reader, with the measurement taken at 24 h intervals for 72 h. Excitation and emission wavelengths of Thioflavin T were 440 and 508 nm, respectively. All assays were performed at least twice to ensure reproducibility.

#### 3.6.3. Stock Preparation of AcPHF6 Peptide

In brief, AcPHF6 peptide was dissolved in 50 mM phosphate buffer (pH 7.4) at a concentration of 1 mM and vortexed to obtain a homogeneous solution. For aggregation inhibition experiments, 100 μM AcPHF6 peptide was mixed with compounds **6** and incubated at 37 °C for 4 days. The degree of AcPHF6 aggregation was determined using Thioflavin-T fluorescence analyses.

#### 3.6.4. AcPHF6 Aggregation Inhibition

Briefly, the screen of the styrylquinolines was performed based on Thioflavin T fluorescence. All assays were done on flat black 96-well plates. For the inhibition of aggregation assays, 100 µM AcPHF6 and 10 µM ThT were incubated in 50 mM phosphate buffer (pH 7.4) containing various concentrations of compounds **6** (0.5, 1, 5, 10 and 20 µM). Immediately before the experiment, 1 μM heparin was added to initiate AcPHF6 aggregation. AcPHF6 alone in buffer reaction served as a control for the screen. Fluorescence values were taken at 24 h intervals for 72 h, at 37 °C and in replicates, using a microplate reader. Measurements with Thioflavin T were performed at excitation and emission wavelengths of 440 and 508 nm, respectively. All assays were performed at least twice to ensure reproducibility.

#### 3.6.5. Cell Line Model

The stable cell line that overexpresses Tau protein (HEK293-Tau3R, Tau 3R isoform) was a kind donation of Dr. Jesús Ávila (Center of Molecular Biology “Severo Ochoa”, CSIC, Universidad Autónoma, Madrid, Spain). Cells were cultured in DMEM medium supplemented with 10% fetal bovine serum, 1 mM pyruvate, 2 mM glutamine and 0.2 mg/mL of the antibiotic zeocin. Cultures were maintained at 37 °C in a humidified atmosphere containing 5% CO_2_.

#### 3.6.6. Cell Viability

HEK293-Tau3R cells were seeded into 96-well polystyrene plates at a density of 20,000 cells per well and incubated at 37 °C for 24 h to allow cell adhesion. The plates were then treated with compounds **6** at concentrations ranging from 0.1 to 10 µM for an additional 24 h. Cell viability was assessed by measuring metabolic activity through the reduction of MTT (3-(4,5-dimethylthiazol-2-yl)-2,5-diphenyltetrazolium bromide) to insoluble formazan. Absorbance values were measured at 550 nm using a microplate reader.

#### 3.6.7. Determination of Caspase-3 Activity on Cells Treated with Okadaic Acid

HEK293-Tau3R cells in logarithmic growth phase were digested with trypsin at a concentration of 0.25% and then resuspended in the medium and subcultured in Petri dishes (100 × 15 mm) at a seeding density of 1.5 × 10^5^ cells/mL. After 24 h, cells were pre-treated with each one of the styrylquinolines (100 nM, non-toxic concentration) for 1 h and then with 25 nM okadaic acid for 24 h, concomitantly with the styrylquinolines. Upon completion of the treatment, cells were collected, washed twice with PBS (pH 7.4) and pelleted by centrifugation at 1000× *g* for 5 min. The pellet was then resuspended in PBS and centrifuged again at 1000× *g* for 10 min at 4 °C to remove the supernatant. The resulting cell pellets were lysed in a buffer containing 10 mM Tris–HCl (pH 7.5) with 0.5% CHAPS, 1 mM MgCl_2_, 1 mM EGTA, 1 mM EDTA, 10% glycerol, 5 mM β-mercaptoethanol, 1 mM DTT, 1 mM phenylmethylsulfonyl fluoride (PMSF), 100 μM leupeptin and 1 μM pepstatin. Following a 30 min incubation on ice, the lysates were clarified by centrifugation at 13,000× *g* for 5 min. Protein concentrations in the supernatants were determined using the bicinchoninic acid (BCA) assay.

The caspase 3 activity assay performed using the known fluorogenic substrate Ac-DEVD-AMC. HEK293-Tau3R cells were harvested, washed with ice-cold PBS and lysed in 100 μL of lysis buffer containing 50 mM HEPES (pH 7.4), 100 mM NaCl, 0.1% CHAPS, 10% glycerol, 10 mM DTT, 0.1 mM EDTA and protease inhibitors (1 μg/μL each of leupeptin, pepstatin and aprotinin). The lysates were, sonicated and centrifuged at 14,000× *g* for 15 min at 4 °C. For the assay, 20 μg of cellular protein from the lysates were incubated with 20 µM caspase 3 substrate in assay buffer (50 mM HEPES, pH 7.4, 100 mM NaCl, 0.1% CHAPS, 10% glycerol, 10 mM DTT) for 2 h at 37 °C. Substrate cleavage was monitored using a microplate fluorescence reader with excitation at 360 nm and emission at 460 nm. Caspase activity was determined based on the cleavage of the fluorogenic substrate and is expressed as fluorescence arbitrary units.

## 4. Conclusions

2-Styrylquinoline derivatives containing an embedded push-pull system, were synthesized and their fluorescent characterization was performed. They change their spectroscopic properties upon protein interaction and show sensitivity to pH and environment polarity, showing red shifts in lower polarity environments. Inner charge transfer phenomena were detected and exploited to detect the interaction of these compounds with protein aggregates. The use of these compounds for the ex vivo staining of β-amyloid plates in samples of cerebral tissue from an Alzheimer patient under fluorescence microscopy, using immunofluorescence techniques, was demonstrated, and shown to be selective over phosphorylated tau. With the present study, we also demonstrated the anti-amyloidogenic effect of the styrylquinolines toward amyloid β (Aβ_1–42_) and AcPHF6 (the hexapeptide ^306^VQIVYK^311^ segment from the microtubule binding region of Tau protein, which is known to promote nucleation dependent Tau-aggregation), both implicated in Alzheimer’s disease. These styrylquinolines significantly inhibited the aggregation in vitro, in a dose-dependent manner as evident from Thioflavin T binding assay, and showed neuroprotective activity following okadaic acid insult. Our findings underscore the dual nature of styrylquinolines as a suitable scaffold for designing novel AD theranostic agents, while uncovering two suitable candidates for fluorescent Aβ imaging in *post-mortem* tissue or animal models (compounds **6b** and **6c**), and another compound (**6h**) suitable as a lead for anti-aggregation therapy development. Additional potential directions for future research are the study of the interaction of compounds **6** with proteins relevant in other proteinopathies and the introduction of structural modifications that shift the emission maxima of compounds **6** further into the NIR region.

## Figures and Tables

**Table 1:** Binding constants values (*K*_d_, μM) and standard deviations obtained for compounds **6** and fibrils of β-amyloid peptide, employing thioflavin T (Th T) as reference.

ThT	6a	6b	6c	6d	6e	6f	6g	6h
16.76 ± 0.84	2.43 ± 0.32	7.93 ± 0.63	8.19 ± 0.61	6.60 ± 0.79	10.20 ± 1.53	1.82 ± 0.25	*	7.01 ± 0.49

* The *K*_d_ value could not be calculated in this case.

## Data Availability

The original contributions presented in this study are included in the article/Supplementary Materials. Further inquiries can be directed to the corresponding author.

## Supplementary Materials

The following supporting information can be downloaded at: https://www.mdpi.com/article/10.3390/ijms26178270/s1.

## Abbreviations

The following abbreviations are used in this manuscript:

AD	Alzheimer’s disease
ALS	Amyotrophic lateral sclerosis
APP/PS1	Amyloid precursor protein/presenilin 1 transgenic mice
BCA	Bicinchoninic acid
CDK5	Cyclin-dependent kinase 5
CERAD	Consortium to establish a registry for Alzheimer’s disease
CHAPS	3-[(3-Cholamidopropyl)dimethylammonio]-1-propanesulfonate
DIBAL	Di-isobutyl aluminum hydride
DMEM	Dulbecco’s modified Eagle medium
DTT	Dithiothreitol
EDTA	Ethylenediaminotetraacetic acid
EGTA	Ethylene glycol-bis(β-aminoethyl ether)-*N*,*N*,*N*′,*N*′-tetraacetic acid tetrasodium salt
FMT	Fluorescent molecular tomography
FMT-CT	Fluorescent molecular tomography/computed tomography
GSK3β	Glycogen synthase kinase 3β
HEK293-Tau3R	Human embryonic kidney 293, containing 3 microtubule binding repeats
HEPES	4-(2-Hydroxyethyl)-1-piperazineethanesulfonic acid
ICT	Intramolecular charge transfer
MTT	3-(4,5-Dimethylthiazol-2-yl)-2,5-diphenyltetrazolium bromide
NIR	Near-infrared
OK	Okadaic acid
PB	Phosphate buffer
PKA	Protein kinase A
PMD	Protein misfolding diseases
PMSF	Phenylmethylsulfonyl fluoride
PP2A	Protein phosphatase 2A
SEM	Standard error of the mean
SPECT	Single-photon emission computed tomography
THF	Tetrahydrofuran
ThS	Thioflavin S
ThT	Thioflavin T
UV-Vis	Ultraviolet-visible

## References

[1] Wang S., Jiang Y., Yang A., Meng F., Zhang J. (2025). The expanding burden of neurodegenerative diseases: An unmet medical and social need. Aging Dis..

[2] Hao M., Chen J. (2025). Trend analysis and future predictions of global burden of Alzheimer’s disease and other dementias: A study based on the global burden of disease database from 1990 to 2021. BMC Med..

[3] Nandi A., Counts N., Chen S., Seligman B., Tortorice D., Vigo D., Bloom D.E. (2022). Global and regional projections of the economic burden of Alzheimer’s disease and related dementias from 2019 to 2050: A value of statistical life approach. eClinicalMedicine.

[4] Luo Y., Qiao L., Li M., Wen X., Zhang W., Li X. (2025). Global, regional, national epidemiology and trends of Parkinson’s disease from 1990 to 2021: Findings from the Global Burden of Disease Study 2021. Front. Aging Neurosci..

[5] Su D., Cui Y., He C., Yin P., Bai R., Zhu J., Lam J.S.T., Zhang J., Yan R., Zheng X. (2025). Projections for prevalence of Parkinson’s disease and its driving factors in 195 countries and territories to 2050: Modelling study of Global Burden of Disease Study 2021. BMJ.

[6] Klug G.M., Wand H., Simpson M., Boyd A., Law M., Masters C.L., Matěj R., Howley R., Farrell M., Breithaupt M. (2013). Intensity of human prion disease surveillance predicts observed disease incidence. J. Neurol. Neurosurg. Psychiatry.

[7] Bertran-Recasens B., Vidal-Notari S., Hernández Guillamet G., López Seguí F., Vidal-Alaball J., Jiménez-Balado J., Rubio M.A. (2025). Epidemiology of amyotrophic lateral sclerosis: A population-based analysis, 2015–2020. Amyotroph. Lateral Scler. Front. Degener..

[8] Cianci V., Cianci A., Sapienza D., Cracò A., Germanà A., Ieni A., Gualniera P., Asmundo A., Mondello C. (2024). Epidemiological changes in transthyretin cardiac amyloidosis: Evidence from in vivo data and autoptic series. J. Clin. Med..

[9] Fereshtehnejad S.-M., Lökk J. (2025). Healthcare complexities in neurodegenerative proteinopathies: A narrative review. Healthcare.

[10] Basha S., Mukunda D.C., Rodrigues J., Gail D’Souza M., Gangadharan G., Pai A.R., Mahato K.K. (2023). A comprehensive review of protein misfolding disorders, underlying mechanism, clinical diagnosis, and therapeutic strategies. Ageing Res. Rev..

[11] Vaquer-Alicea J., Diamond M.I. (2019). Propagation of protein aggregation in neurodegenerative diseases. Annu. Rev. Biochem..

[12] Khan A.N., Khan R.H. (2022). Protein misfolding and related human diseases: A comprehensive review of toxicity, proteins involved, and current therapeutic strategies. Int. J. Biol. Macromol..

[13] Chen Y., Liu Q., Yang F., Yu H., Xie Y., Yao W. (2022). Lysozyme amyloid fibril: Regulation, application, hazard analysis, and future perspectives. Int. J. Biol. Macromol..

[14] Das M., Gursky O. (2015). Amyloid-forming properties of human apolipoproteins: Sequence analyses and structural insights. Adv. Exp. Med. Biol..

[15] Bhowmick D.C., Kudaibergenova Z., Burnett L., Jeremic A.M. (2022). Molecular mechanisms of amylin turnover, misfolding and toxicity in the pancreas. Molecules.

[16] Wang L., Hall C.E., Uchikawa E., Chen D., Choi E., Zhang X., Bai X.-C. (2023). Structural basis of insulin fibrillation. Sci. Adv..

[17] Rajan R., Ahmed S., Sharma N., Kumar N., Debas A., Matsumura K. (2021). Review of the current state of protein aggregation inhibition from a materials chemistry perspective: Special focus on polymeric materials. Mater. Adv..

[18] Aliyan A., Cook N.P., Martí A.A. (2019). Interrogating amyloid aggregates using fluorescent probes. Chem. Rev..

[19] Giorgetti S., Greco C., Tortorella P., Aprile F.A. (2018). Targeting amyloid aggregation: An overview of strategies and mechanisms. Int. J. Mol. Sci..

[20] Eisele Y.S., Monteiro C., Fearns C., Encalada S.E., Wiseman R.L., Powers E.T., Kelly J.W. (2015). Targeting protein aggregation for the treatment of degenerative diseases. Nat. Rev. Drug Discov..

[21] Zhang X., Wang J., Zhang Z., Ye K. (2024). Tau in neurodegenerative diseases: Molecular mechanisms, biomarkers, and therapeutic strategies. Transl. Neurodegener..

[22] Iqbal K., Liu F., Gong C.X. (2016). Tau and neurodegenerative disease: The story so far. Nat. Rev. Neurol..

[23] Jack C.R., Knopman D.S., Jagust W.J., Petersen R.C., Weiner M.W., Aisen P.S., Shaw L.M., Vemuri P., Wiste H.J., Weigand S.D. (2013). Tracking pathophysiological processes in Alzheimer’s disease: An updated hypothetical model of dynamic biomarkers. Lancet Neurol..

[24] Buchhave P., Minthon L., Zetterberg H., Wallin Å.K., Blennow K., Hansson O. (2012). Cerebrospinal fluid levels of β-amyloid 1–42, but not of tau, are fully changed already 5 to 10 years before the onset of Alzheimer dementia. JAMA Psychiatry.

[25] Pawlowski M., Meuth S.G., Duning T. (2017). Cerebrospinal fluid biomarkers in Alzheimer’s disease—From brain metabolism to clinical application. Diagnostics.

[26] Luo Z., Xu H., Liu L., Ohulchanskyy T.Y., Qu J. (2021). Optical imaging of beta-amyloid plaques in Alzheimer’s disease. Biosensors.

[27] Gao M., Tang B.Z. (2017). Fluorescent sensors based on aggregation-induced emission: Recent advances and perspectives. ACS Sens..

[28] Leite J.P., Figueira F., Mendes R.F., Paz F.A.A., Gales L. (2023). Metal–organic frameworks as sensors for human amyloid diseases. ACS Sens..

[29] Jiang H. (2023). Fluorescence Molecular Tomography. Principles and Applications.

[30] Ntziachristos V. (2010). Going deeper than microscopy: The optical imaging frontier in biology. Nat. Methods.

[31] Li D., Hu Z., Zhang H., Yang Q., Zhu L., Liu Y., Yu T., Zhu J., Wu J., He J. (2022). A Through-Intact-Skull (TIS) chronic window technique for cortical structure and function observation in mice. eLight.

[32] Koronyo Y., Salumbides B.C., Black K.L., Koronyo-Hamaoui M. (2012). Alzheimer’s disease in the retina: Imaging retinal amyloid plaques for early diagnosis and therapy assessment. Neurodegener. Dis..

[33] Schön C., Hoffmann N.A., Ochs S.M., Burgold S., Filser S., Steinbach S., Seeliger M.W., Arzberger T., Goedert M., Kretzschmar H.A. (2012). Long-term in vivo imaging of fibrillar tau in the brains of tauopathy mice using high-resolution large-field multifocal illumination fluorescence microscopy. PLoS ONE.

[34] Gu J., Anumala U.R., Heyny-von Haußen R., Hölzer J., Goetschy-Meyer V., Mall G., Hilger I., Czech C., Schmidt B. (2013). Design, synthesis and biological evaluation of trimethine cyanine dyes as fluorescent probes for the detection of tau fibrils in Alzheimer’s disease brain and olfactory epithelium. ChemMedChem.

[35] Zhang Y., Chen H., Li R., Sterling K., Song W. (2023). Amyloid β-based therapy for Alzheimer’s disease: Challenges, successes and future. Signal Transduct. Target. Ther..

[36] Ghosh S., Ali R., Verma S. (2023). Aβ-oligomers: A potential therapeutic target for Alzheimer’s disease. Int. J. Biol. Macromol..

[37] Sanghai N., Vuong B., Berk A.B., Afridi M.S.K., Tranmer G.K. (2024). Current small molecule–based Medicinal Chemistry approaches for neurodegeneration therapeutics. ChemMedChem.

[38] Miller S., Blanco M.J. (2021). Small molecule therapeutics for neuroinflammation-mediated neurodegenerative disorders. RSC Med. Chem..

[39] Ramsay R.R., Popovic-Nikolic M.-R., Nikolic K., Uliassi E., Bolognesi M.L. (2018). A perspective on multi-target drug discovery and design for complex diseases. Clin. Trans. Med..

[40] Kabir A. (2022). Polypharmacology: The science of multi-targeting molecules. Pharmacol. Res..

[41] Maddeboina K., Yada B., Kumari S., McHale C., Pal D., Durden D.L. (2024). Recent advances in multitarget-directed ligands via in silico drug discovery. Drug Discov. Today.

[42] Hong Y., Meng L., Chen S., Leung C.W., Da L.T., Faisal M., Silva D.A., Liu J., Lam J.W., Huang X. (2012). Monitoring and inhibition of insulin fibrillation by a small organic fluorogen with aggregation-induced emission characteristics. J. Am. Chem. Soc..

[43] Staderini M., Aulić S., Bartolini M., Tran H.N., González-Ruiz V., Pérez D.I., Cabezas N., Martínez A., Martín M.A., Andrisano V. (2012). A fluorescent styrylquinoline with combined therapeutic and diagnostic activities against Alzheimer’s and prion diseases. ACS Med. Chem. Lett..

[44] Wang X.Q., Xia C.L., Chen S.B., Tan J.H., Ou T.M., Huang S.L., Li D., Gu L.Q., Huang Z.S. (2015). Design, synthesis, and biological evaluation of 2-arylethenylquinoline derivatives as multifunctional agents for the treatment of Alzheimer’s disease. Eur. J. Med. Chem..

[45] Musiol R. (2020). Styrylquinoline—A versatile scaffold in medicinal chemistry. Med. Chem..

[46] Dhanawat M., Mehta D.K., Das R. (2021). An elite scaffold and a wonderful pharmacophore in drug discovery: Styrylquinoline. Mini Rev. Med. Chem..

[47] Yang Y., Jia H.M., Liu B.L. (2012). (*E*)-5-Styryl-1*H*-indole and (*E*)-6-styrylquinoline derivatives serve as probes for β-amyloid plaques. Molecules.

[48] Burkett B.J., Bartlett D.J., McGarrah P.W., Lewis A.R., Johnson D.R., Berberoğlu K., Pandey M.K., Packard A.T., Halfdanarson T.R., Hruska C.B. (2023). A review of theranostics: Perspectives on emerging approaches and clinical advancements. Radiol. Imaging Cancer.

[49] Pratihar S., Bhagavath K.K., Govindaraju T. (2023). Small molecules and conjugates as theranostic agents. RSC Chem. Biol..

[50] Bongarzone S., Staderini M., Bolognesi M.L. (2014). Multitarget ligands and theranostics: Sharpening the medicinal chemistry sword against prion diseases. Future Med. Chem..

[51] Sarabia-Vallejo A., López-Alvarado P., Menéndez J.C. (2023). Small-molecule theranostics in Alzheimer’s disease. Eur. J. Med. Chem..

[52] Staderini M., Cabezas N., Bolognesi M.L., Menéndez J.C. (2011). A general protocol for the solvent-and catalyst-free synthesis of 2-styrylquinolines under focused microwave irradiation. Synlett.

[53] Vedamalai M., Krishnakumar V.G., Gupta S., Mori S., Gupta I. (2017). Synthesis and characterization of styryl-BODIPY derivatives for monitoring in vitro Tau aggregation. Sens. Actuators B.

[54] Ranee S.J., Sivaraman G., Pushpalatha A.M., Muthusubramanian S. (2018). Quinoline based sensors for bivalent copper ions in living cells. Sens. Actuators B.

[55] Ziaunys M., Sakalauskas A., Mikalauskaite K., Smirnovas V. (2021). Polymorphism of alpha-synuclein amyloid fibrils depends on ionic strength and protein concentration. Int. J. Mol. Sci..

[56] Carapeto A.P., Marcuello C., Faísca P.F.N., Rodrigues M.S. (2024). Morphological and biophysical study of S100A9 protein fibrils by atomic force microscopy imaging and nanomechanical analysis. Biomolecules.

[57] Rajasekhar K., Narayanaswamy N., Murugan N.A., Kuang G., Ågren H., Govindaraju T. (2016). A high affinity red fluorescence and colorimetric probe for amyloid aggregates. Sci. Rep..

[58] Morais G.R., Miranda H.V., Santos I.C., Santos I., Outeiro T.F., Paulo A. (2011). Synthesis and in vitro evaluation of fluorinated styryl benzazoles as amyloid-probes. Bioorg. Med. Chem..

[59] Lockhart A., Ye L., Judd D.B., Merritt A.T., Lowe P.N., Morgenstern J.L., Hong G., Gee A.D., Brown J. (2005). Evidence for the presence of three distinct binding sites for the thioflavin T class of Alzheimer’s disease PET imaging agents on β-amyloid peptide fibrils. J. Biol. Chem..

[60] Li Q., Min J., Ahn Y.-H., Namm J., Kim E.M., Lui R., Kim H.Y., Ji Y., Wu H., Wisniewski T. (2007). Styryl-based compounds as potential in vivo imaging agents for β-amyloid plaques. ChemBioChem.

[61] Santa-María I., Pérez M., Hernández F., Ávila J., Moreno F.J. (2006). Characteristics of the binding of thioflavin S to tau paired helical filaments. J. Alzheimers Dis..

[62] Cores A., Carmona-Zafra N., Martín-Cámara O., Sánchez J.D., Duarte P., Villacampa M., Bermejo-Bescós P., Martín-Aragón S., León R., Menéndez J.C. (2022). Curcumin-piperlongumine hybrids with a multitarget profile elicit neuroprotection in in vitro models of oxidative stress and hyperphosphorylation. Antioxidants.

[63] Ganguly A., Babu S.S., Ghosh S., Velyutham R., Kapusetti G. (2025). Advances and future trends in the detection of beta-amyloid: A comprehensive review. Med. Eng. Phys..

[64] Kell D.B., Pretorius E. (2017). Proteins behaving badly. Substoichiometric molecular control and amplification of the initiation and nature of amyloid fibril formation: Lessons from and for blood clotting. Prog. Biophys. Mol. Biol..

[65] Mohamed T., Hoang T., Jelokhani-Niaraki M., Rao P.P.N. (2013). Tau-Derived-Hexapeptide ^306^VQIVYK^311^ aggregation inhibitors: Nitrocatechol moiety as a pharmacophore in drug design. ACS Chem. Neurosci..

[66] Ye L., Morgenstern J.L., Lamb J.R., Lockhart A. (2006). Characterisation of the binding of amyloid imaging tracers to rodent Aβ fibrils and rodent-human Aβ co-polymers. Biochem. Biophys. Res. Commun..

[67] Regalado-Reyes M., Furcila D., Hernández F., Ávila J., DeFelipe J., León-Espinosa G. (2019). Phospho-tau changes in the human CA1 during Alzheimer’s disease progression. J. Alzheimers Dis..

