# Supplementary material for: 2-Styrylquinolines with Push-Pull Architectures as Sensors for β-Amyloid Aggregation with Theranostic Properties
Source: Int J Mol Sci. 2025 Aug 26;26(17):8270. doi: 10.3390/ijms26178270 (PMC12428582; doi:10.3390/ijms26178270)
Supplement: Supplementary file 1 [file ijms-26-08270-s001.zip › ijms-3826785-supplementary.pdf]

Supplementary Materials

2-Styrylquinolines with push-pull architectures as sensors for β-amyloid aggregation with theranostic properties

Marta Piquero,[1] Álvaro Sarabia-Vallejo,[1] Latoya Bote-Matías,[2] Gonzalo León-Espinosa,[3] Macarena Hernández-Arasti,[4] Sagrario Martín-Aragón,[4] Paloma Bermejo-Bescós,[4] Ana I. Olives,[2] Pilar López-Alvarado,[1] M. Antonia Martín[2] and J. Carlos Menéndez[1,*]

[1] Unidad de Química Orgánica y Farmacéutica, Departamento de Química en Ciencias Farmacéuticas, Facultad de Farmacia, Universidad Complutense, Plaza de Ramón y Cajal s/n, 28040 Madrid, Spain; e-mail: mpiqueromarti@gmail.com; alsarabi@ucm.es; alvarado@ucm.es.

[2] Unidad de Química Analítica. Departamento de Química en Ciencias Farmacéuticas, Facultad de Farmacia, Universidad Complutense, Plaza de Ramón y Cajal s/n, 28040 Madrid, Spain; e-mail: ariesgen@gmail.com; aiolives@ucm.es; mantonia@farm.ucm.es

[3] Departamento de Química y Bioquímica, Facultad de Farmacia, Universidad San Pablo-CEU, CEU Universities, Urbanización Montepríncipe, 28660, Boadilla del Monte, Madrid, Spain; e-mail: gonzalo.leonespinosa@ceu.es.

[4] Departamento de Farmacología, Farmacognosia y Botánica, Facultad de Farmacia, Universidad Complutense, Plaza de Ramón y Cajal s/n, 28040 Madrid, Spain; e-mail: macarenaarasti@gmail.com; smartina@ucm.es; bescos@ucm.es;

* Correspondence: josecm@farm.ucm.es

Table of contents

Copies of NMR spectra S2

Additional figures S13

Additional tables S20

1. Copies of NMR spectra

(2-Methylquinolin-6-yl)methanol (3)

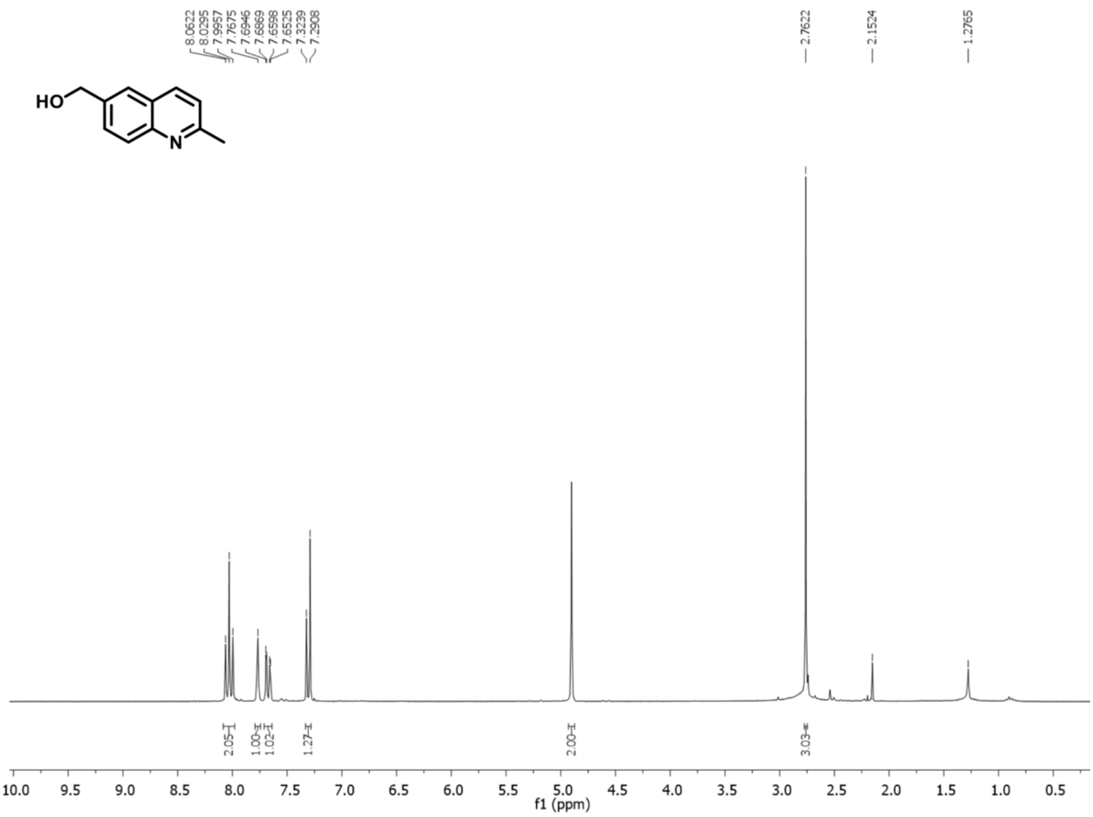

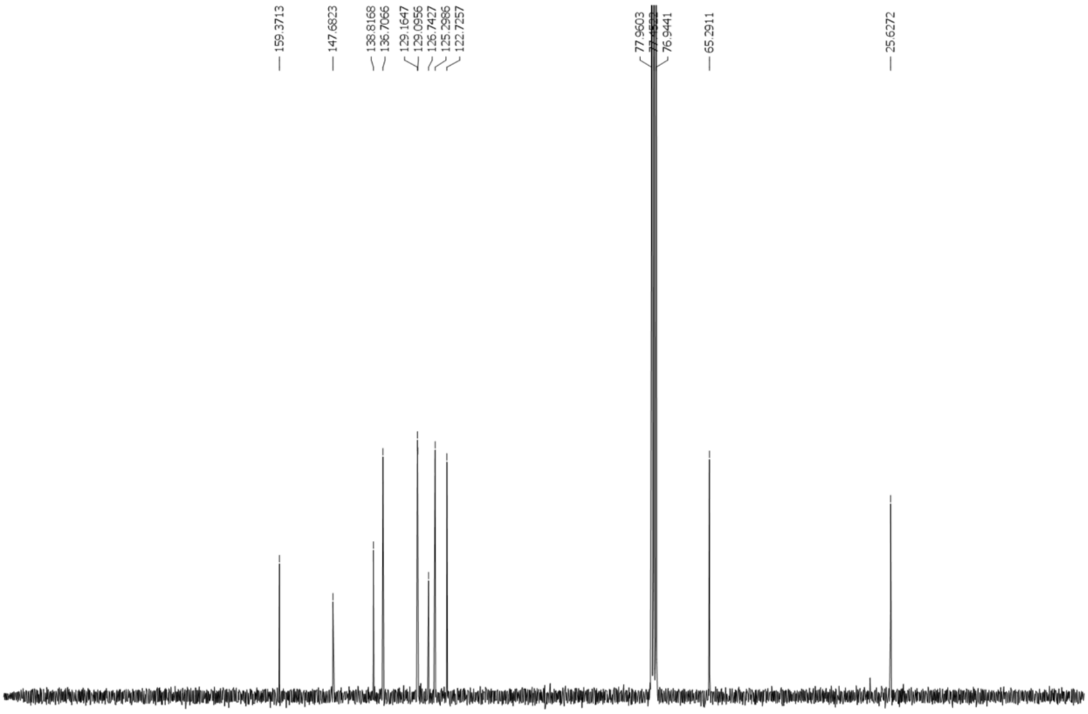

2-Methylquinoline-6-carbaldehyde (4)

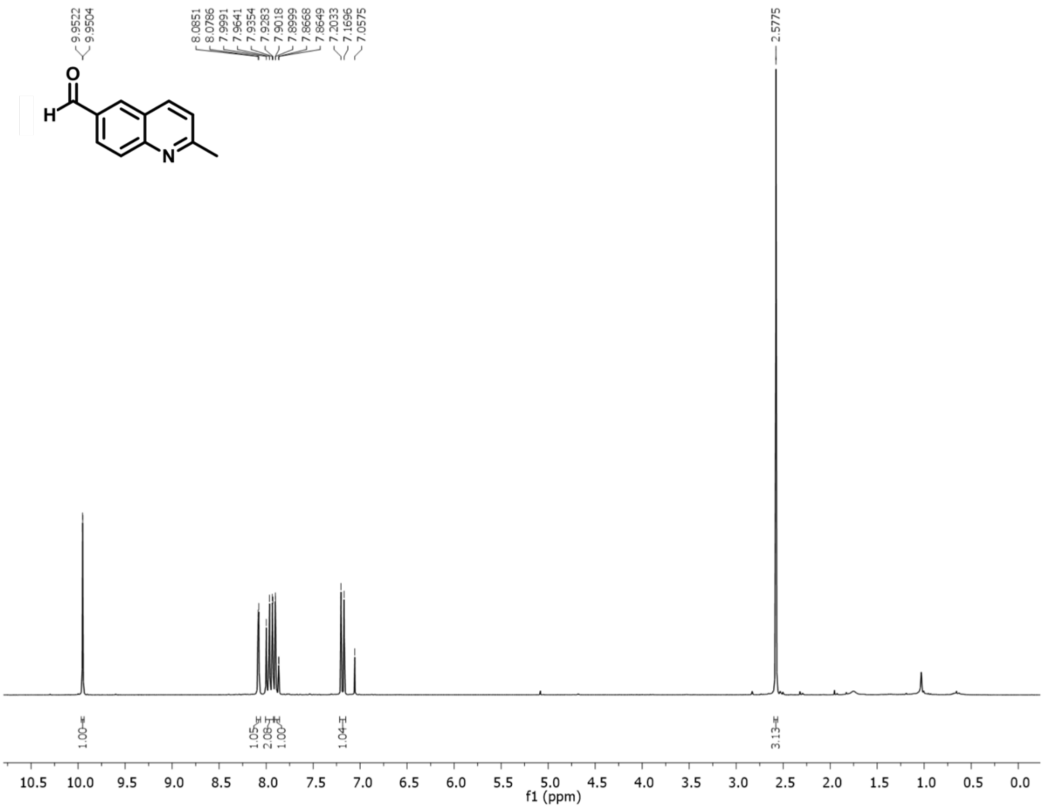

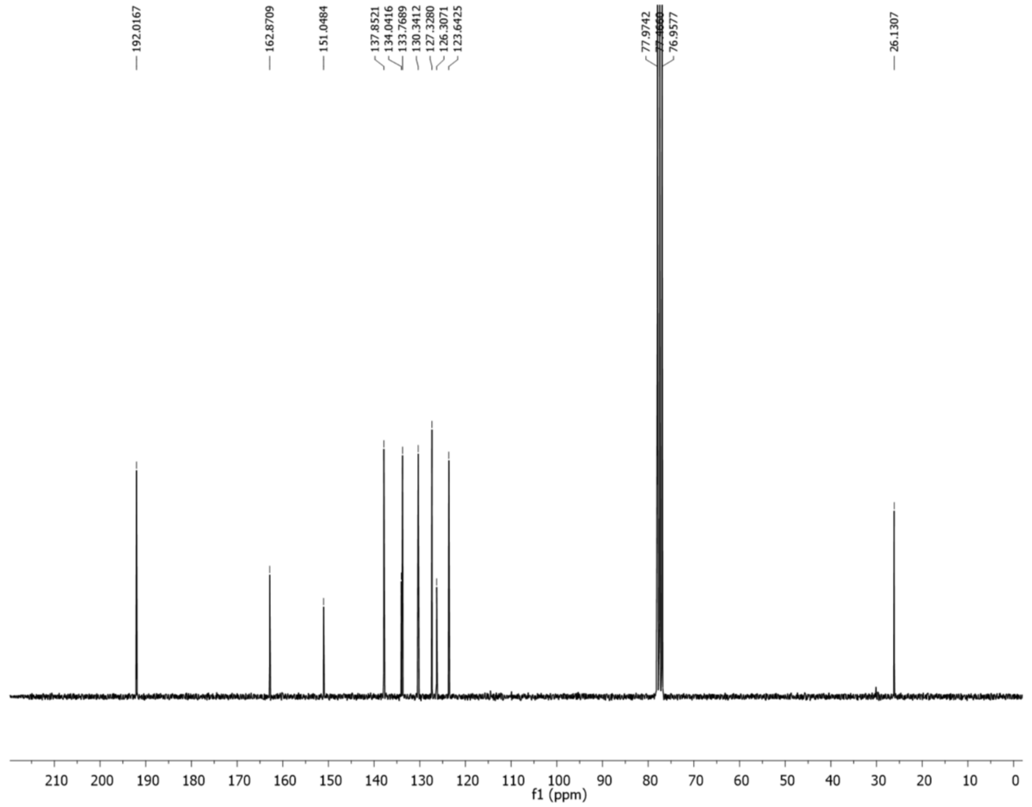

2-((2 Methylquinolin-6-yl)methylene)malononitrile (5)

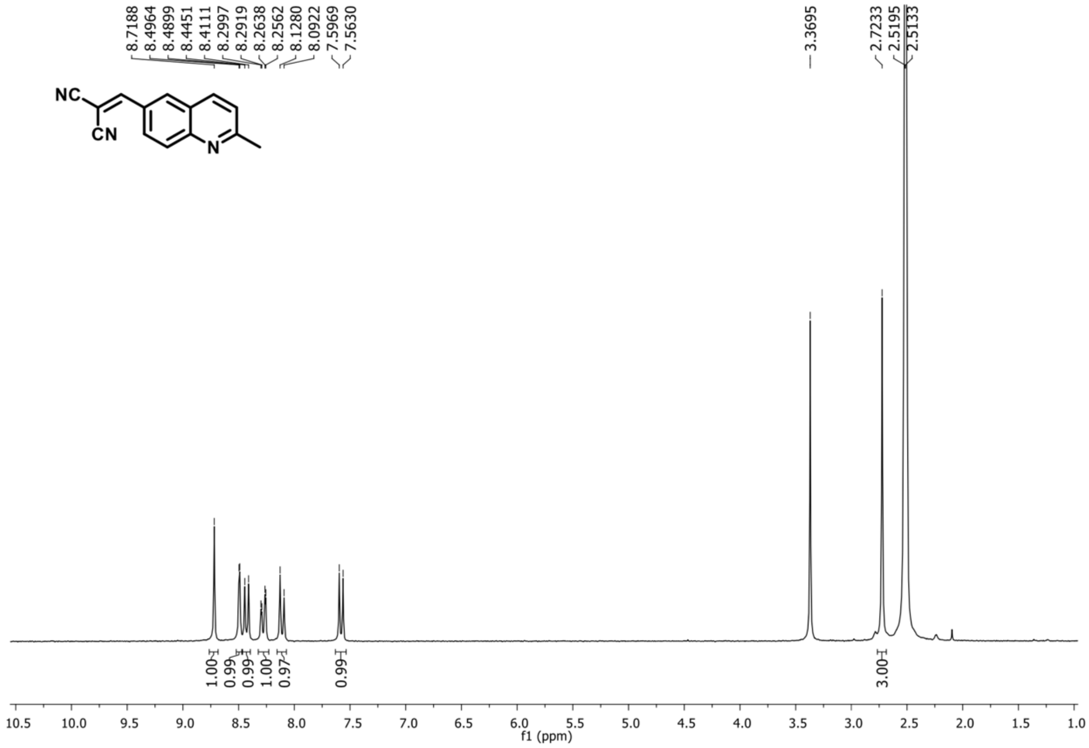

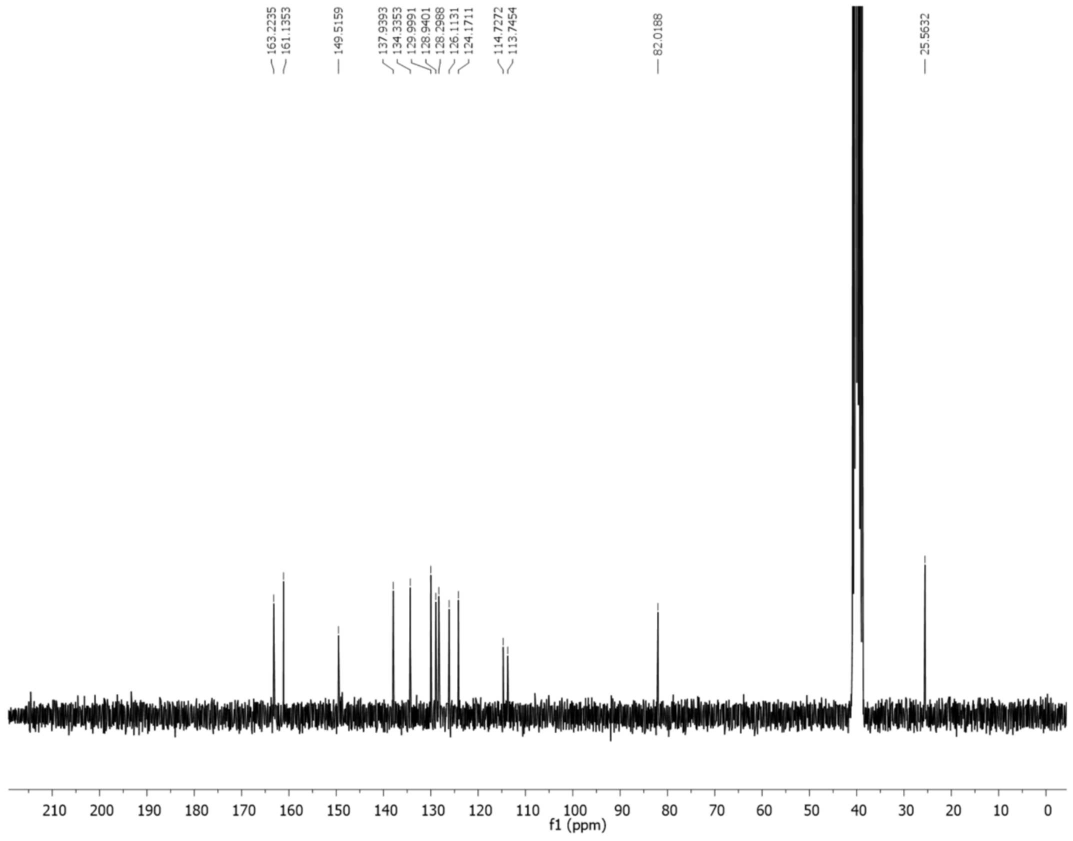

(*E*)-2-((2-(4-(Dimethylamino)styryl)quinolin-6-yl)methylene)malononitrile (6a)

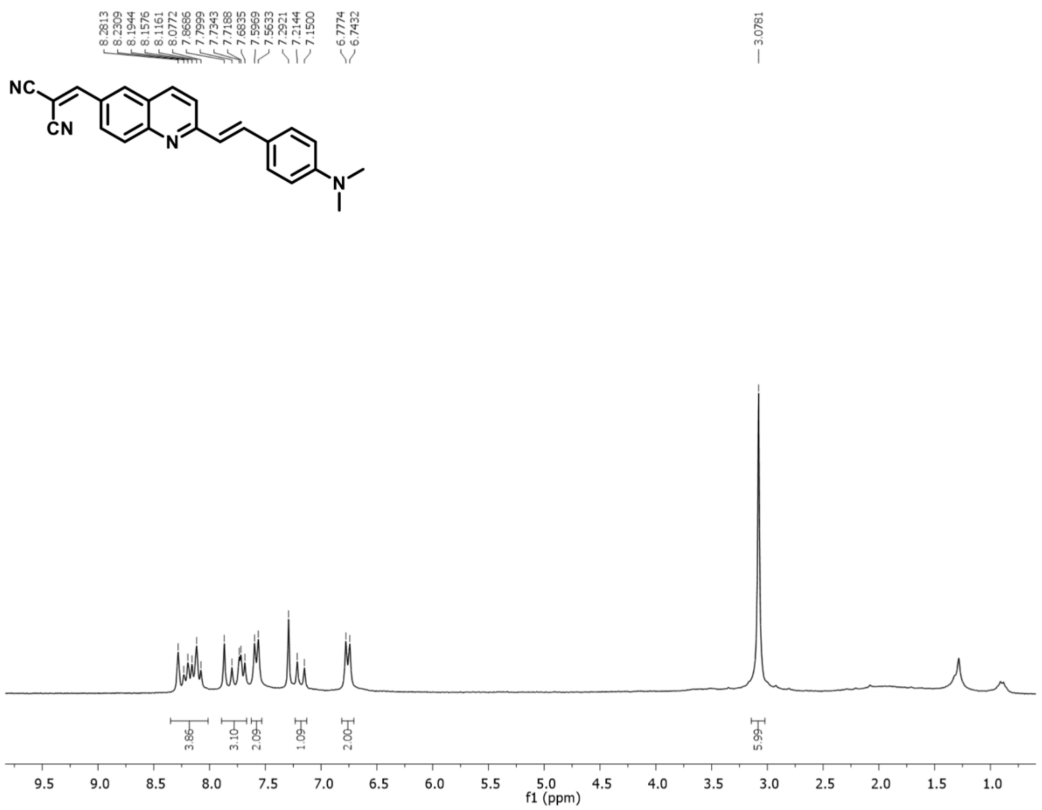

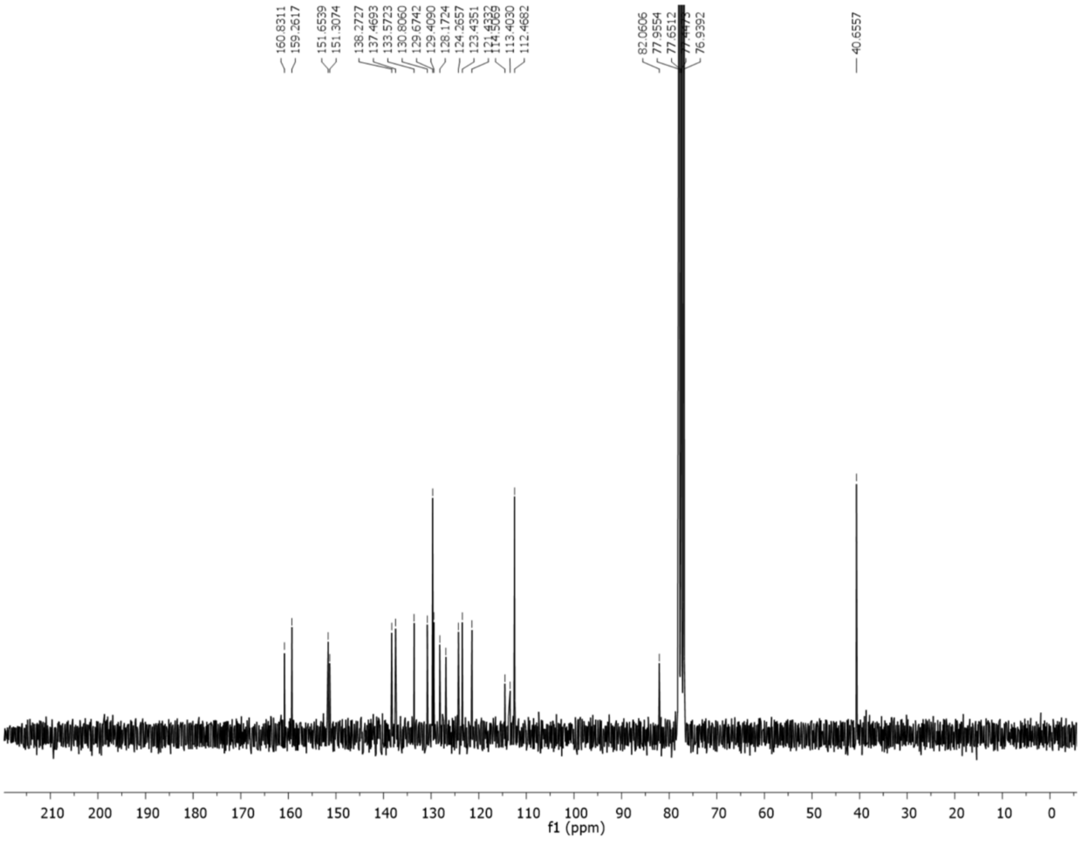

(*E*)-2-((2-(4-(Diethylamino)styryl)quinolin-6-yl)methylene)malononitrile (6b)

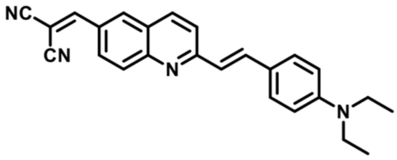

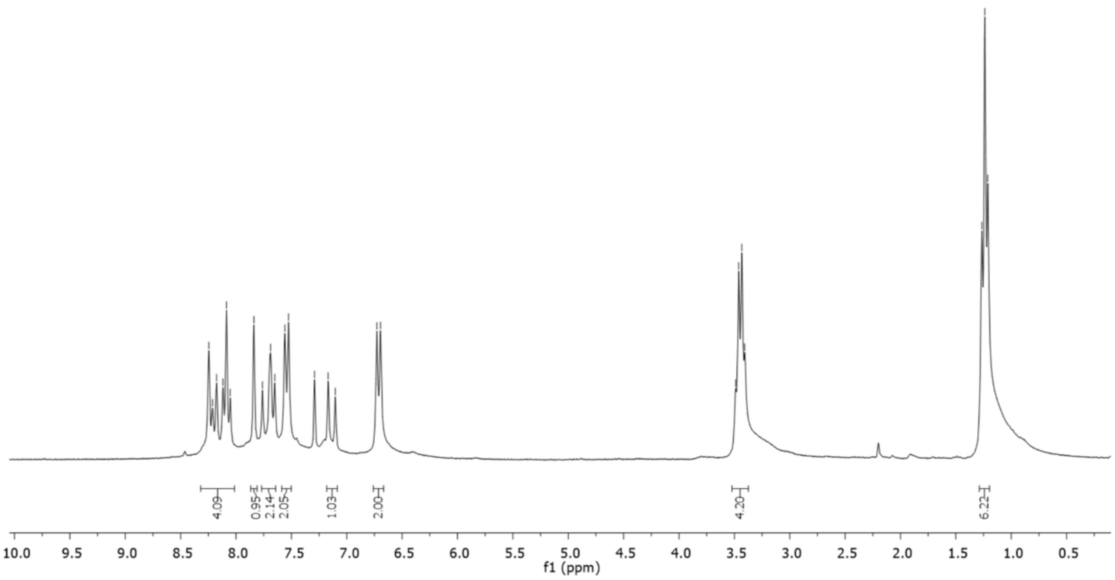

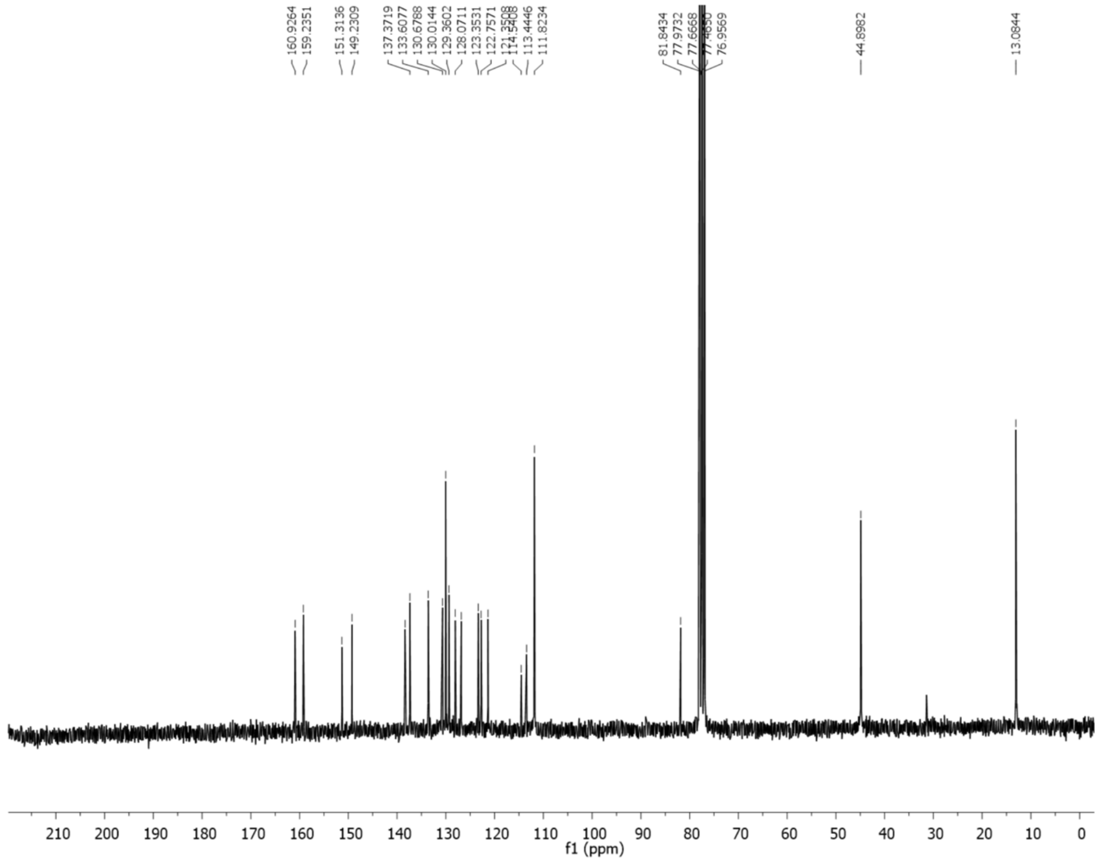

(*E*)-2-((2-(4-(Piperidin-1-yl)styryl)quinolin-6-yl)methylene)malononitrile (6c)

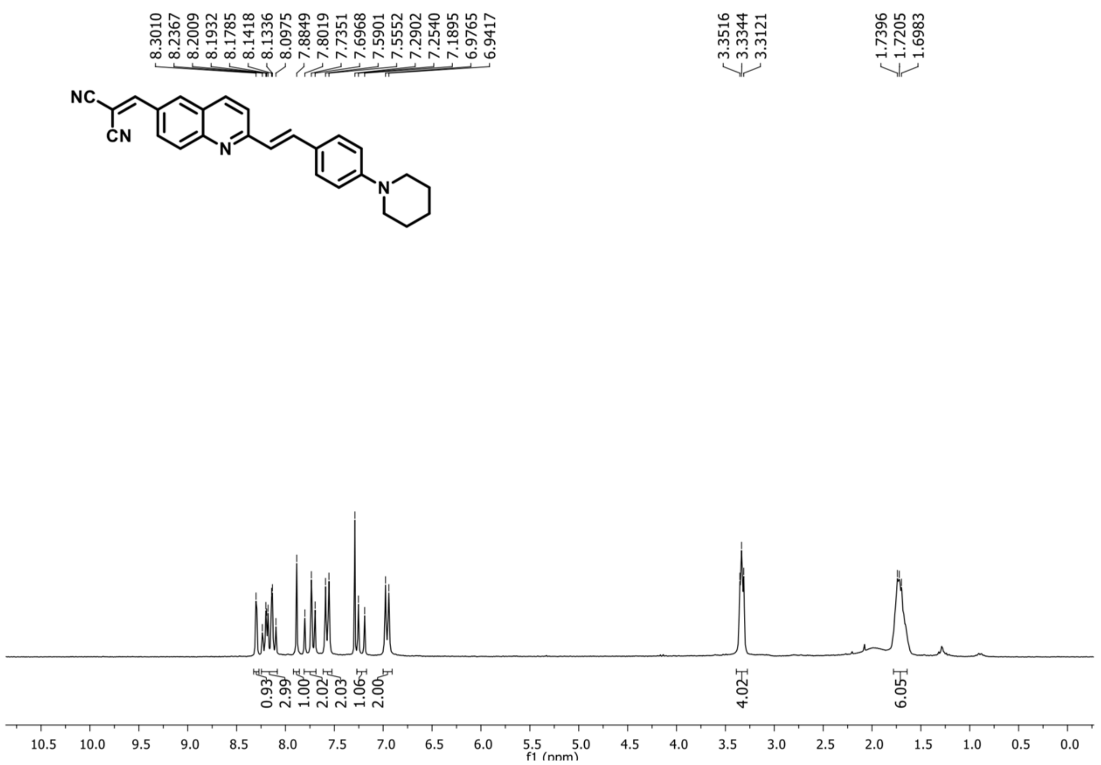

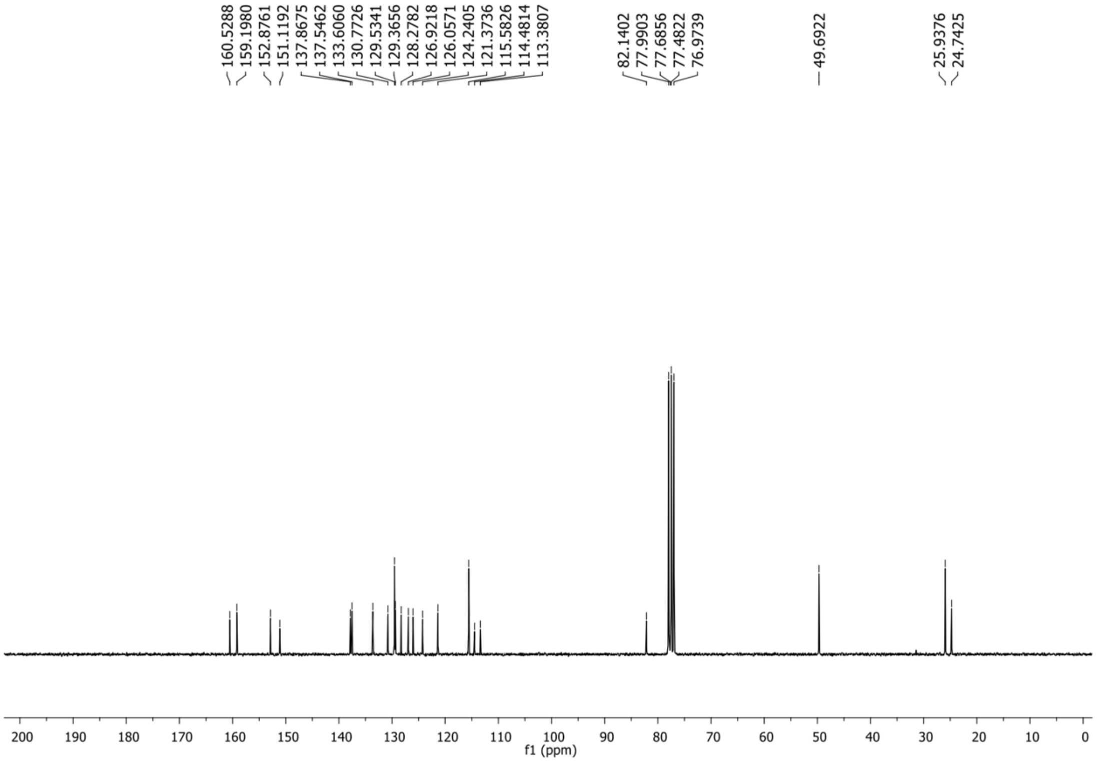

(*E*)-2-((2-(4-Morpholinostyryl)quinolin-6-yl)methylene)malononitrile (6d)

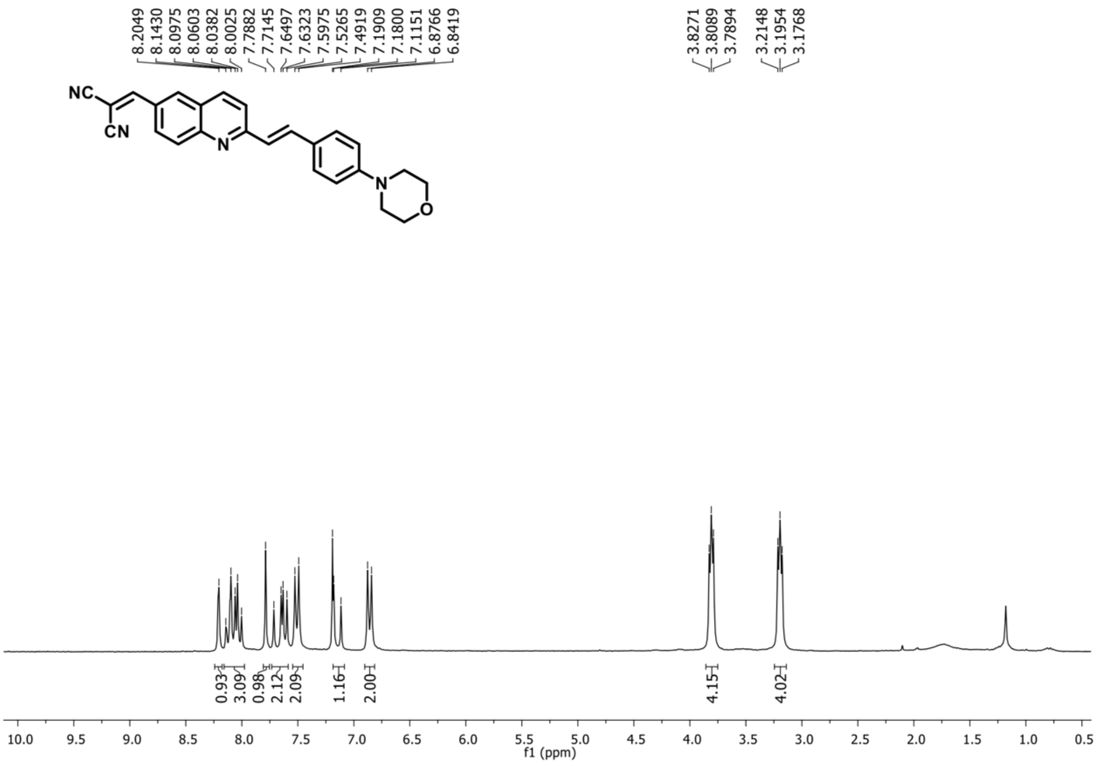

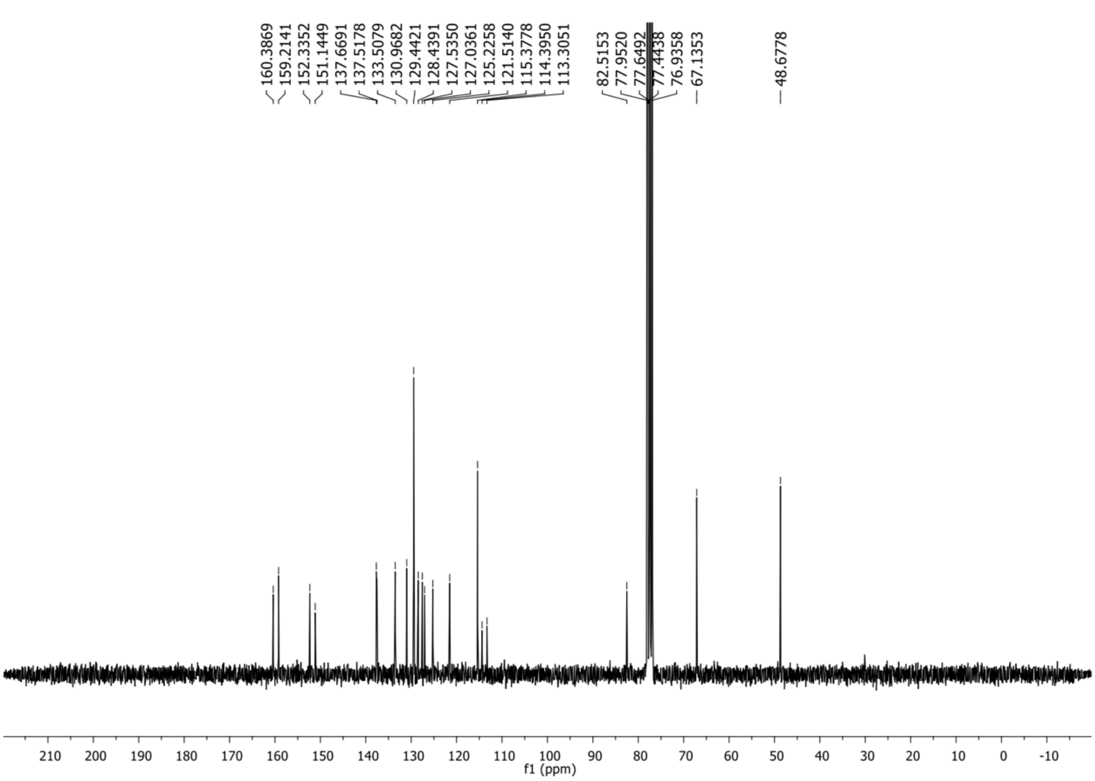

(*E*)-((4-(2-(6-(2,2-Dicyanovinyl)quinolin-2-yl)vinyl)phenyl)azanediyl)-bis(ethane-2,1-diyl) diacetate (6e)

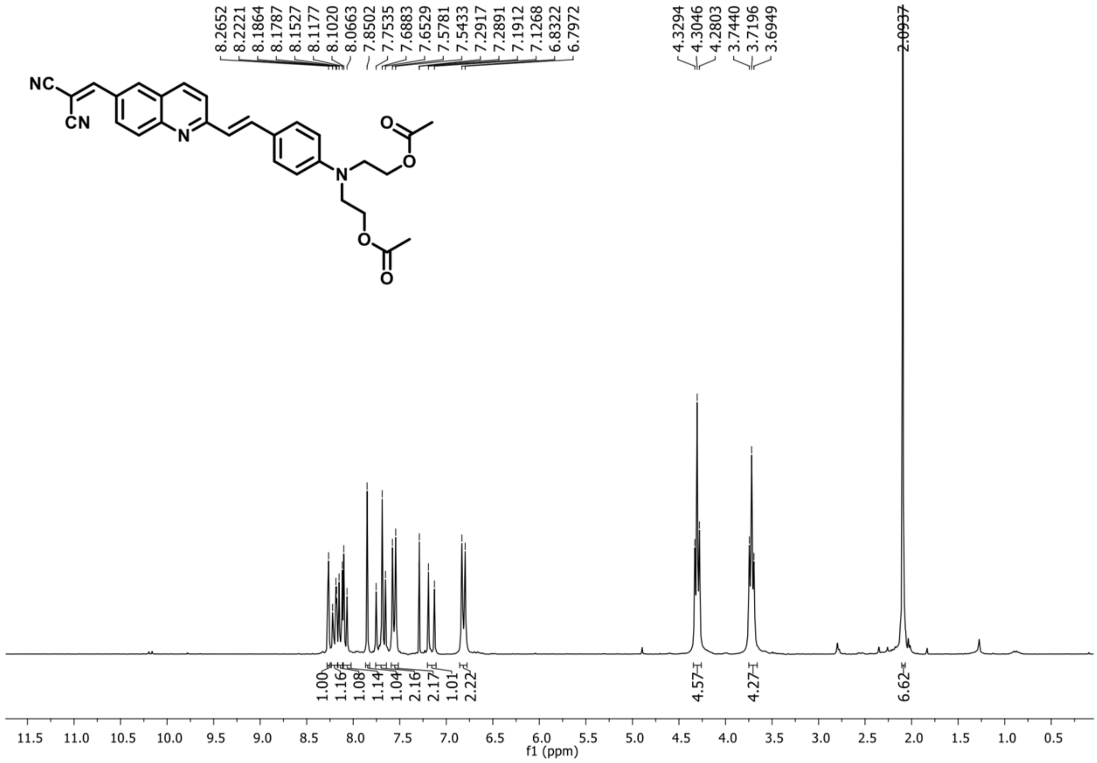

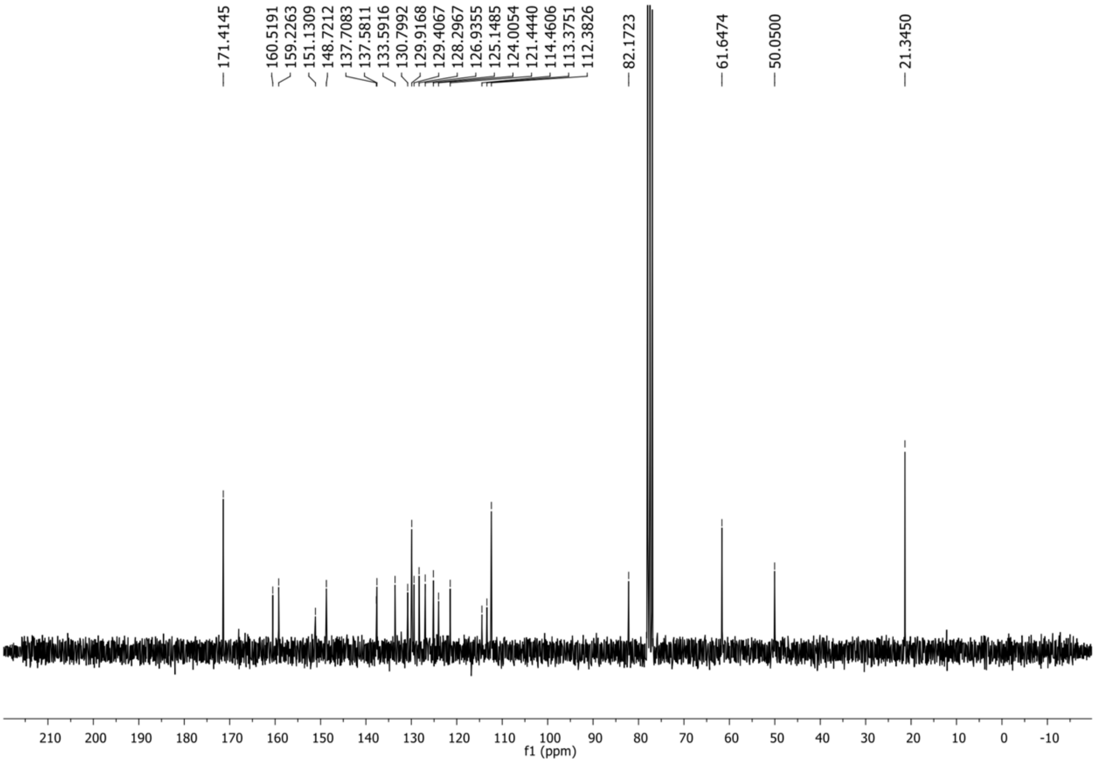

(*E*)-9-(2-(6-(2,2-Dicyanovinyl)quinolin-2-yl)vinyl)-2,3,6,7-tetrahydro-1*H*,5*H*-pyrido[3,2,1-*ij*]quinolin-8-yl acetate (6f)

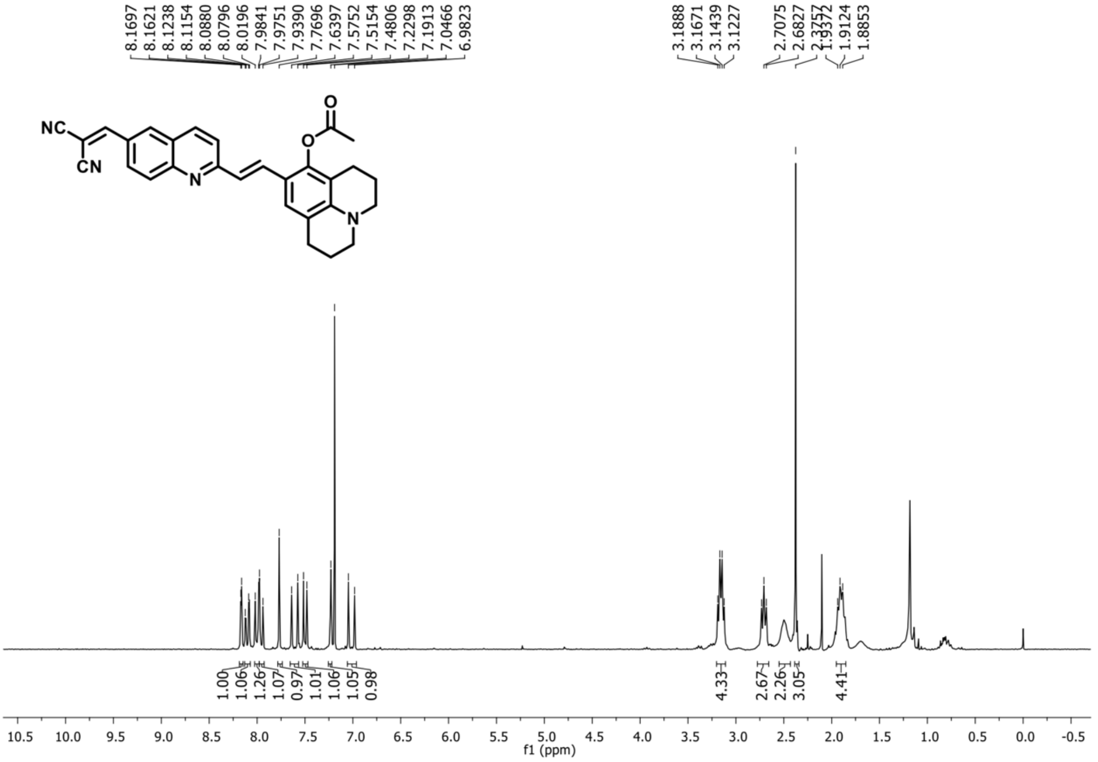

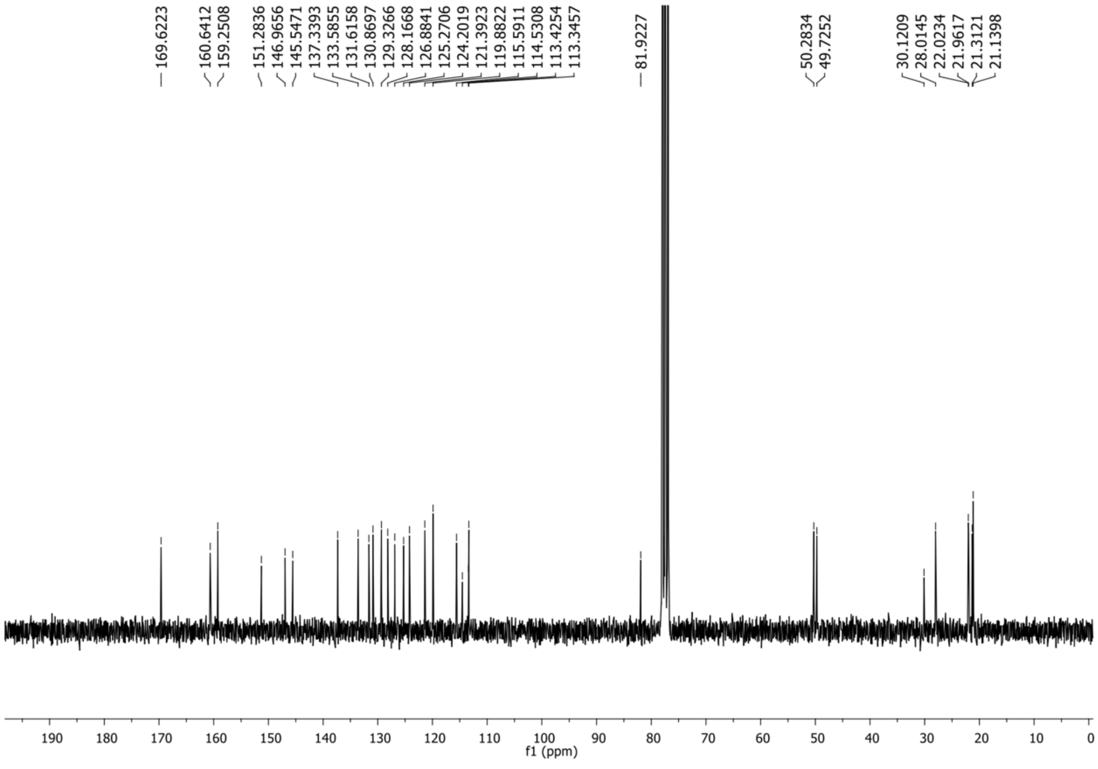

(*E*)-2-((2-(2-(4-(Dimethylamino)naphthalen-1-yl)vinyl)quinolin-6-yl)-methylene)malononitrile (6g)

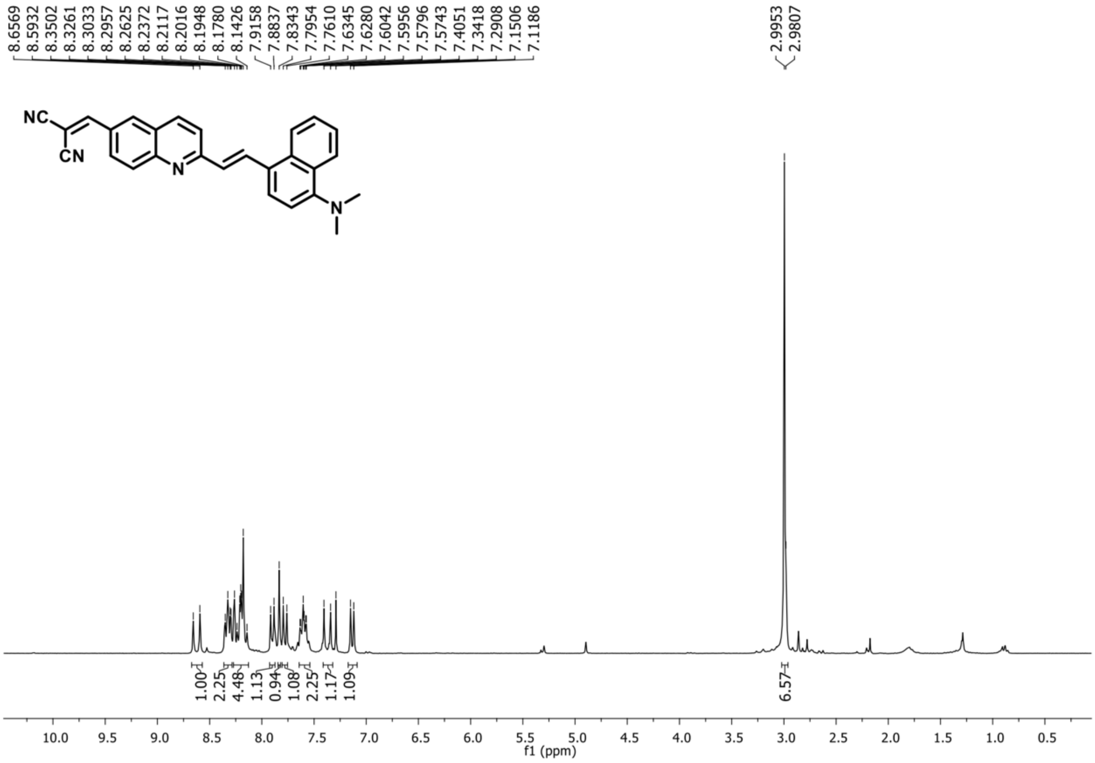

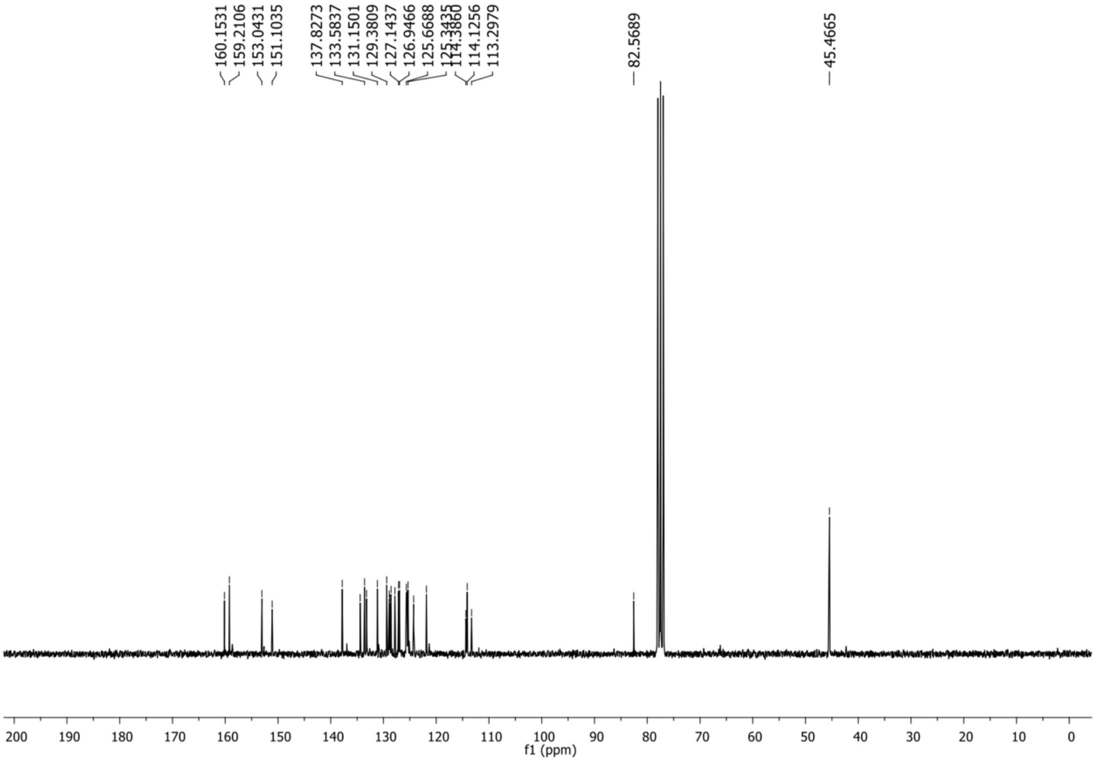

(*E*)-2-((2-(4-(Pyrrolidin-1-yl)styryl)quinolin-6-yl)methylene)malononitrile (6h)

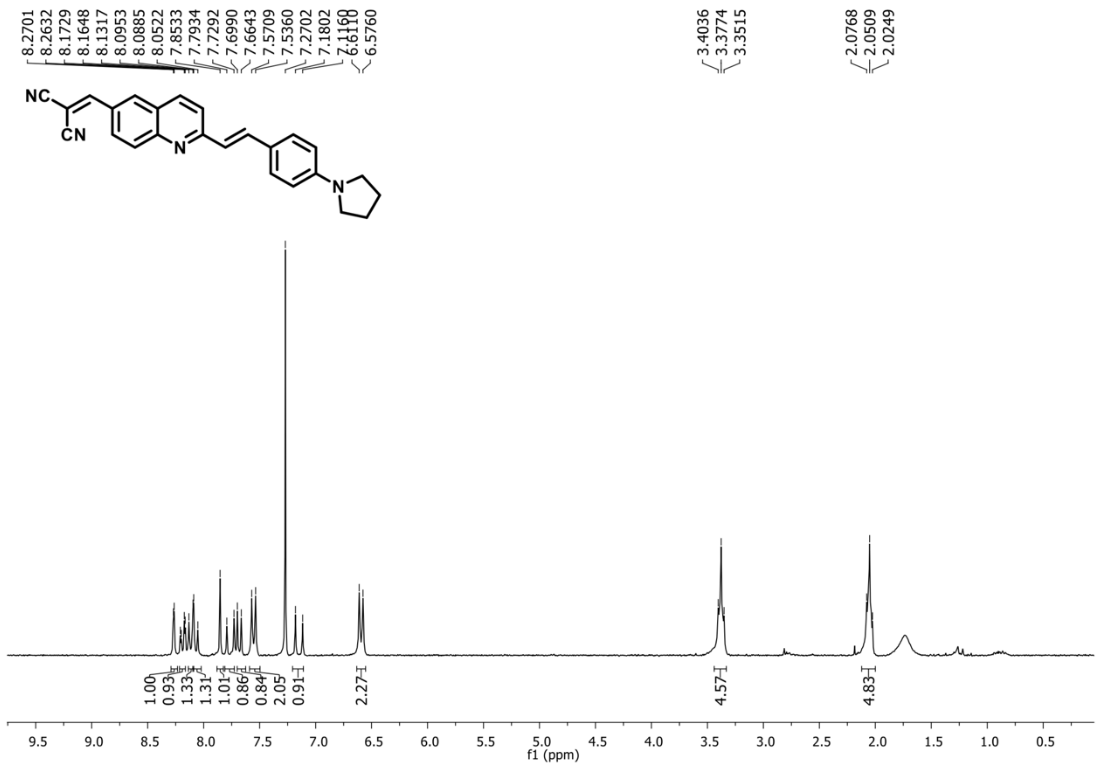

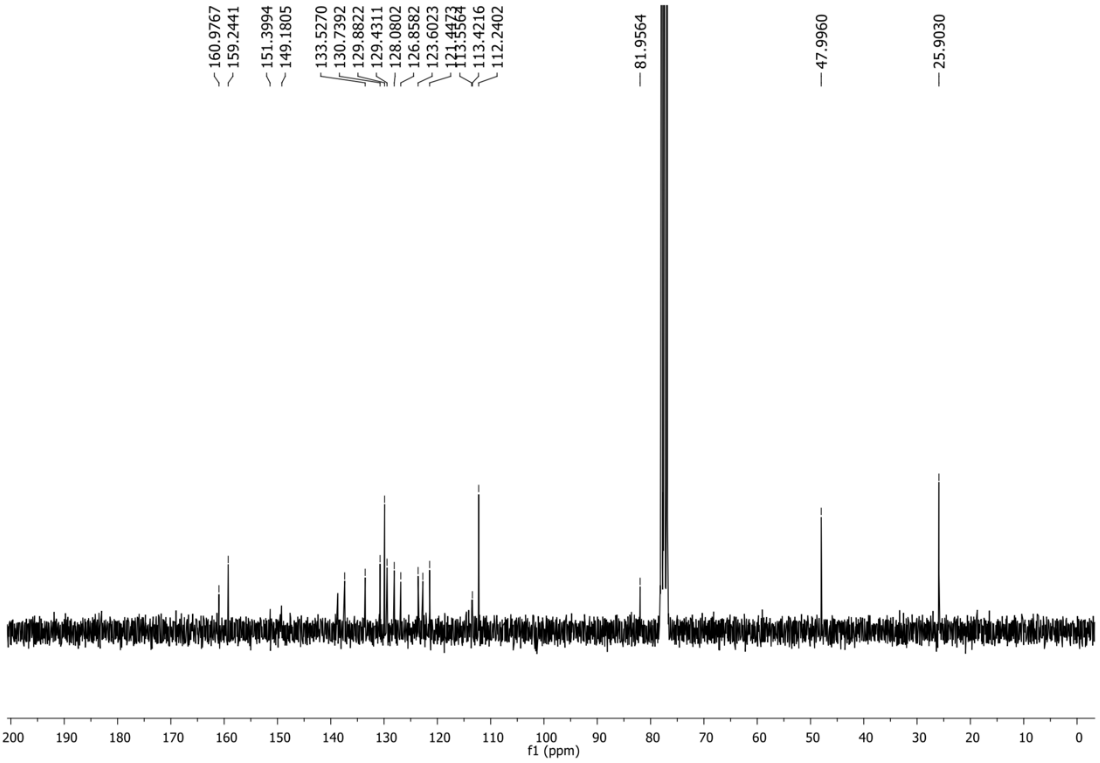

2. Additional Figures

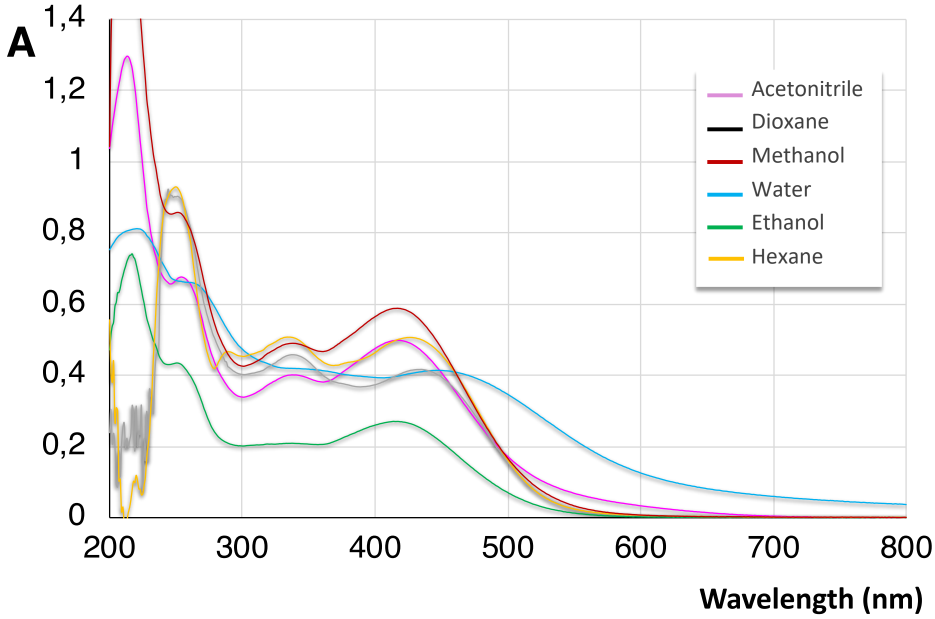

Figure S1. UV-VIS absorption spectra of compound **6h** (1.0×10^{-5} M) in several solvents (A, absorbance).

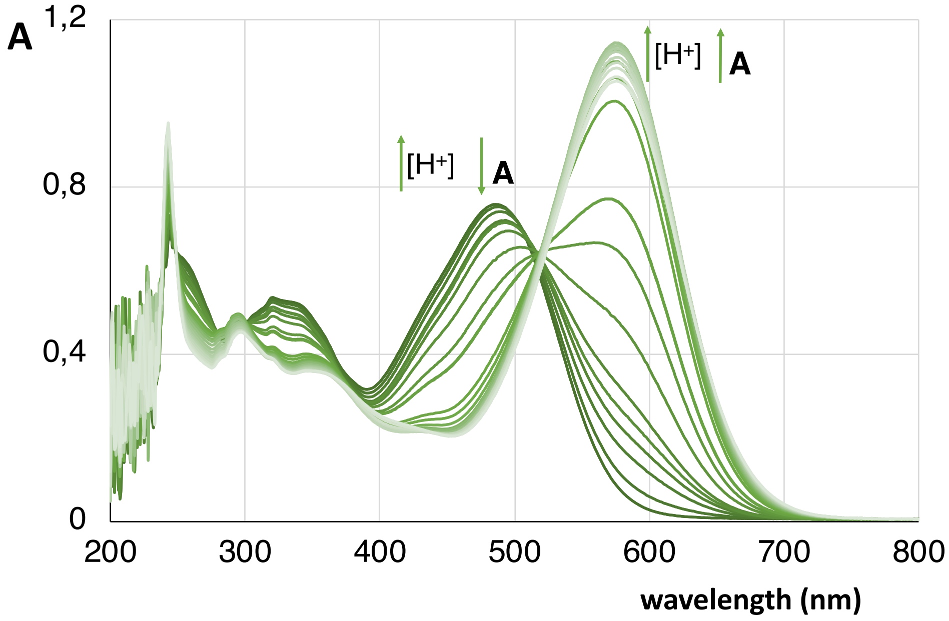

Figure S2. Effect of increasing HCl concentration on the UV-VIS absorption spectra of compound **6f** (5.0×10^{-5} M) in dioxane.

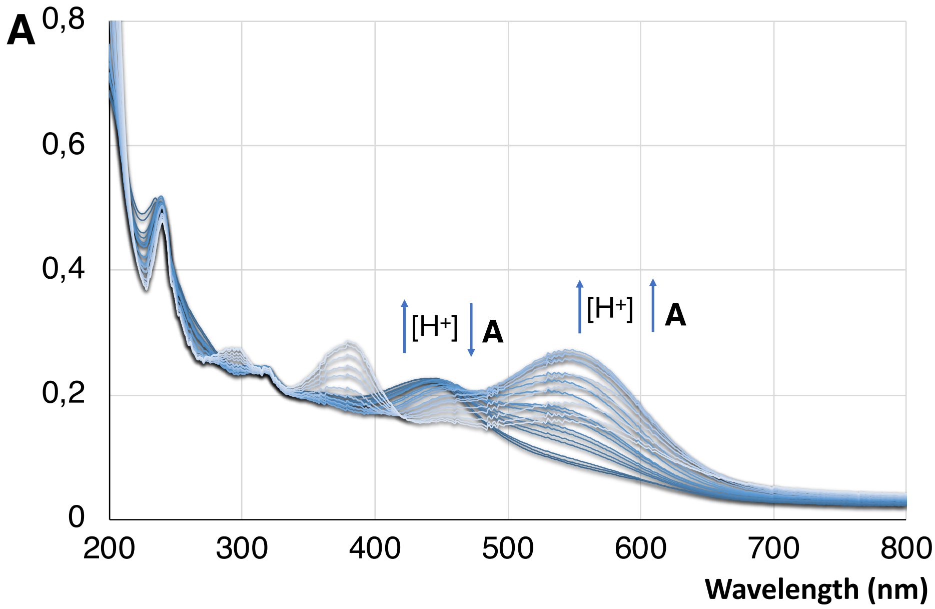

Figure S3. Effect of increasing HCl concentration on the UV-VIS absorption spectra of compound **6f** (5.0×10^{-5} M) in water.

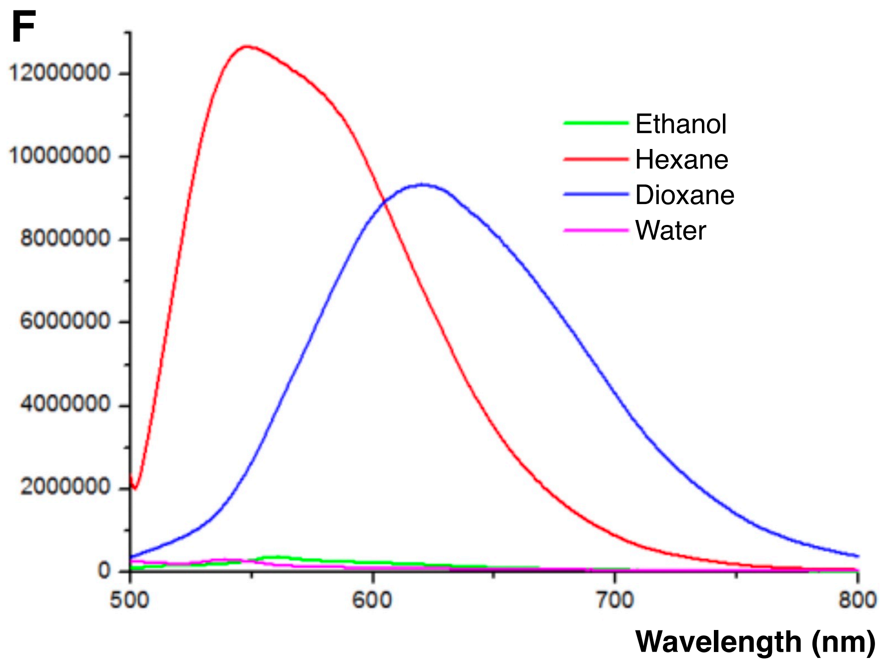

Figure S4. Fluorescence emission spectra of compound **6b** in solvents with varying polarity. Concentrations: 2.5×10^{-7} M for hexane and dioxane (λ_{ex}= 488 nm); 1.0×10^{-6} M for ethanol (λ_{ex}= 480 nm); 1.0×10^{-6} M for water (λ_{ex}= 450 nm). F: Fluorescence in arbitrary units.

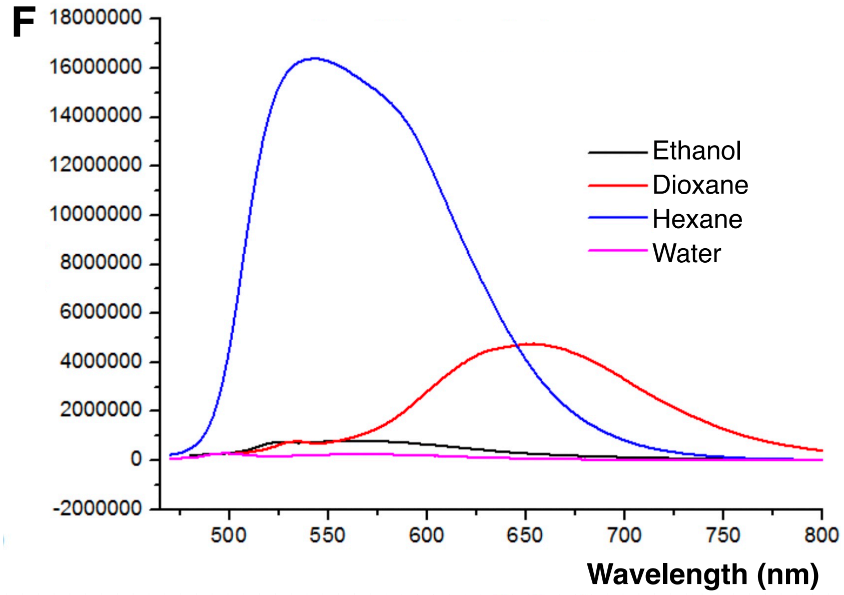

Figure S5. Fluorescence emission spectra of compound **6c** in solvents with varying polarity. Concentrations: 2.5 x 10^{-7} M for hexane and dioxane (λ_{ex}= 450 nm); 1.0 x 10^{-6} M for ethanol (λ_{ex}= 450 nm); 1.0 x 10^{-6} M for water (λ_{ex}= 430 nm). F: Fluorescence in arbitrary units.

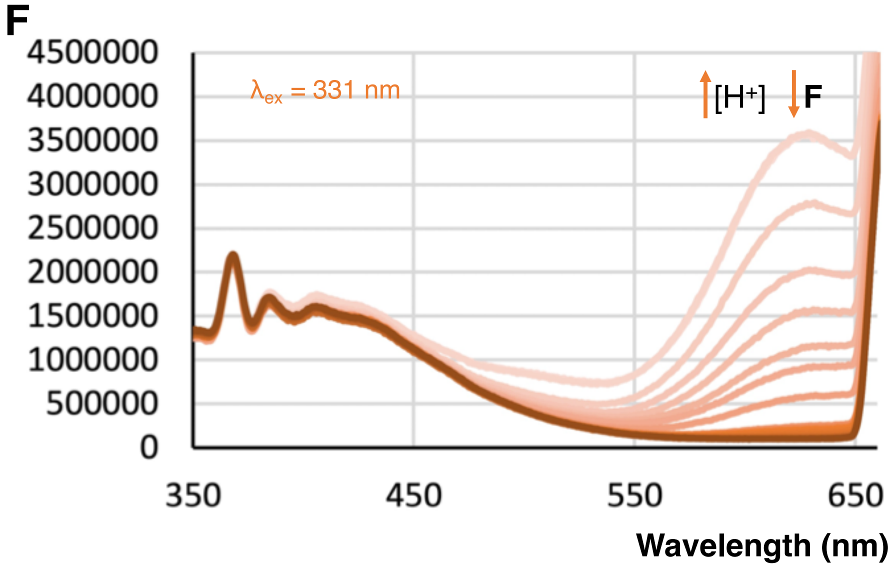

Figure S6. Changes in the fluorescence emission spectra of compound **6a**, 2.5 x 10^{-7} M (λ_{ex}= 331 nm) in dioxane and in the presence of increasing amounts of HCl. F: Fluorescence in arbitrary units.

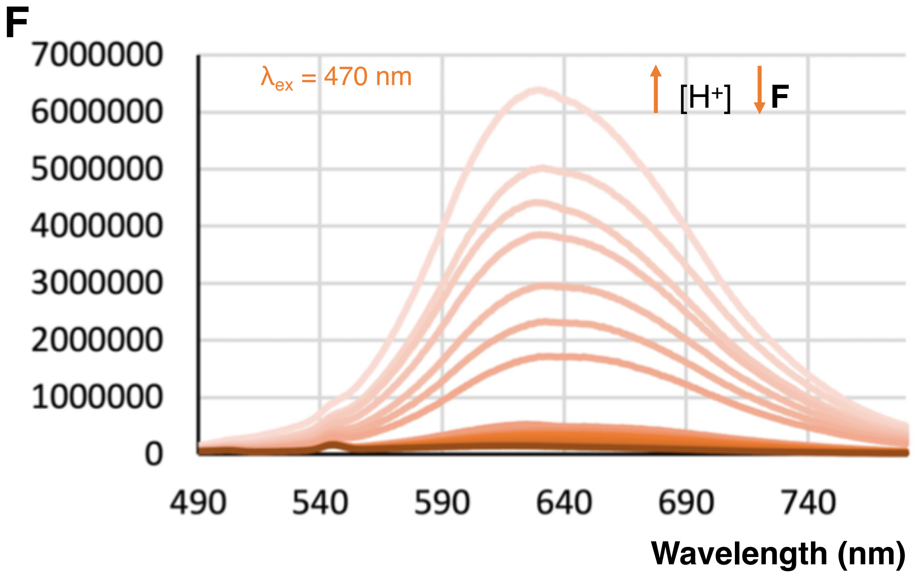

Figure S7. Changes in the fluorescence emission spectra of compound **6a**, 2.5 x 10^{-7} M (λ_{ex}= 470 nm) in dioxane and in the presence of increasing amounts of HCl. F: Fluorescence in arbitrary units.

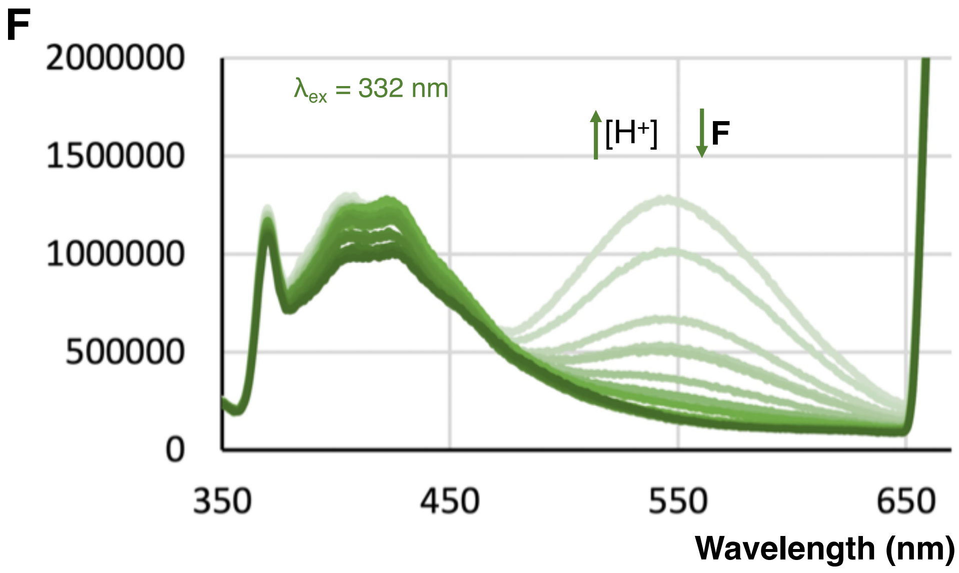

Figure S8. Changes in the fluorescence emission spectra of compound **6a**, 1.0 x 10^{-6} M (λ_{ex}= 332 nm) in ethanol and in the presence of increasing amounts of HCl. F: Fluorescence in arbitrary units.

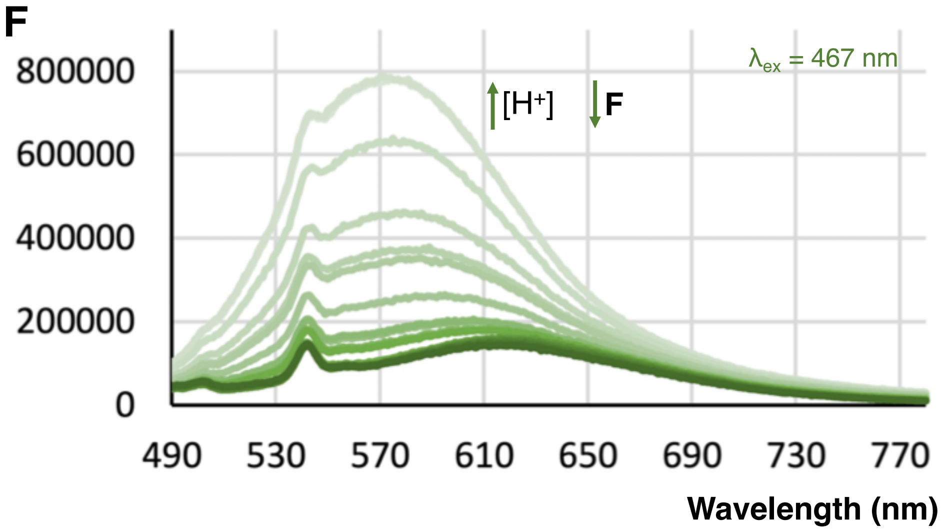

Figure S9. Changes in the fluorescence emission spectra of compound **6a**, 1.0 x 10^{-6} M (λ_{ex}= 467 nm) in ethanol and in the presence of increasing amounts of HCl. F: Fluorescence in arbitrary units.

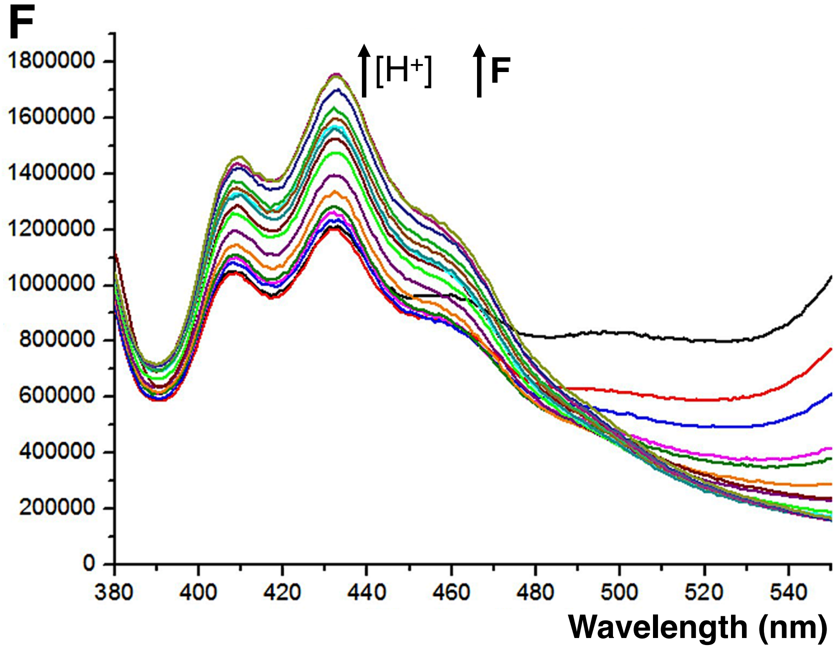

Figure S10. Changes in the fluorescence emission spectra of compound **6d**, 2.5 x 10^{-7} M (λ_{ex}= 335 nm) in dioxane and in the presence of increasing amounts of HCl. F: Fluorescence in arbitrary units.

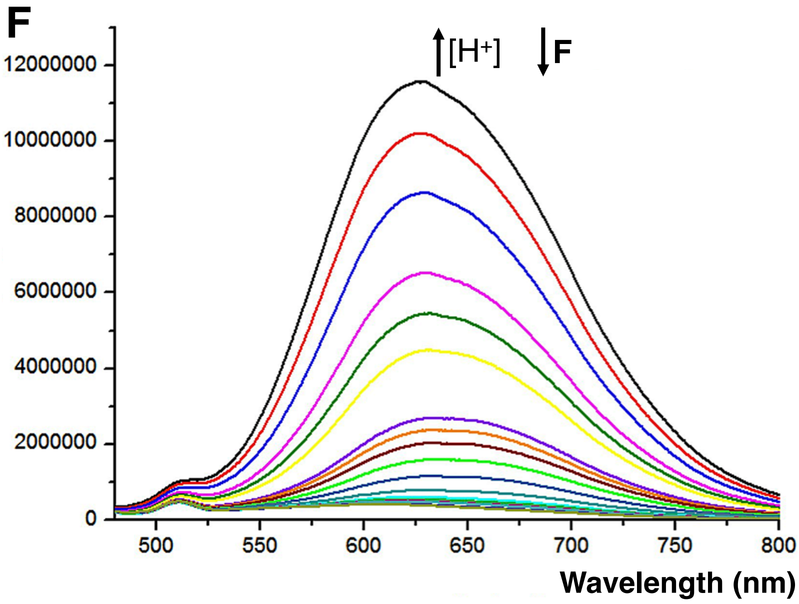

Figure S11. Changes in the fluorescence emission spectra of compound **6d**, 2.5 x 10^{-7} M (λ_{ex}= 445 nm) in dioxane and in the presence of increasing amounts of HCl. F: Fluorescence in arbitrary units.

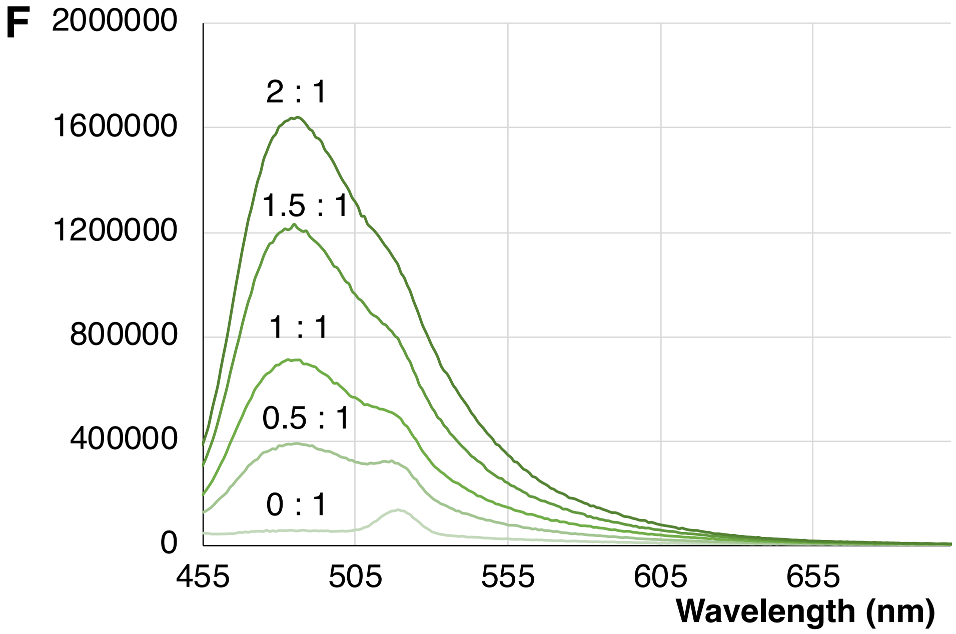

Figure S12. Titration of Thioflavin T (ThT) with increasing concentrations of amyloid fibrils of human β-amyloid protein (fragment 1-42) at the excitation wavelength λ_{ex}= 447 nm. ThT concentration 1.0 x 10^{-6} M. The protein:sensor molar ratios are shown over each spectra. F: Fluorescence in arbitrary units.

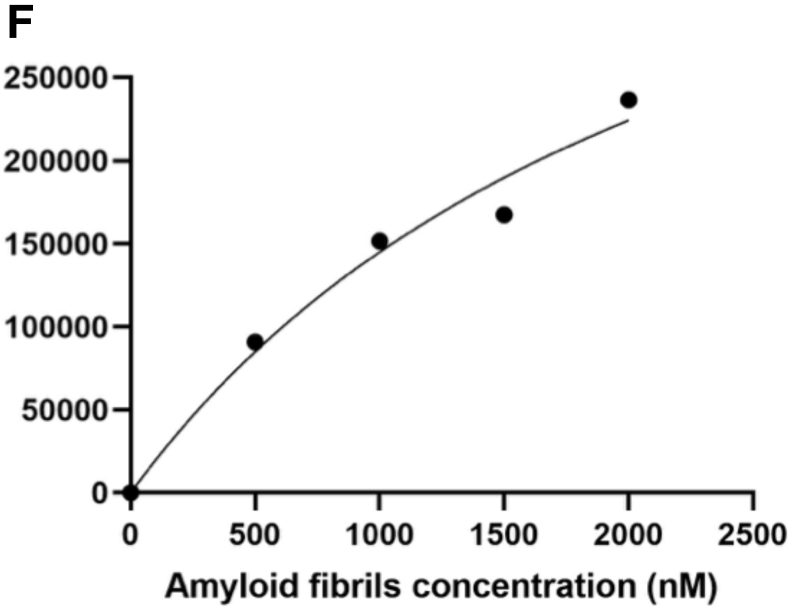

Figure S13. Dissociation constant determination, based on the variation (increasement) of the fluorescence intensity (**F**) of compound **6a** in the presence of increasing concentrations of fibrils of β-amyloid protein. λ_{ex}= 470 nm, λ_{em}= 600 nm.

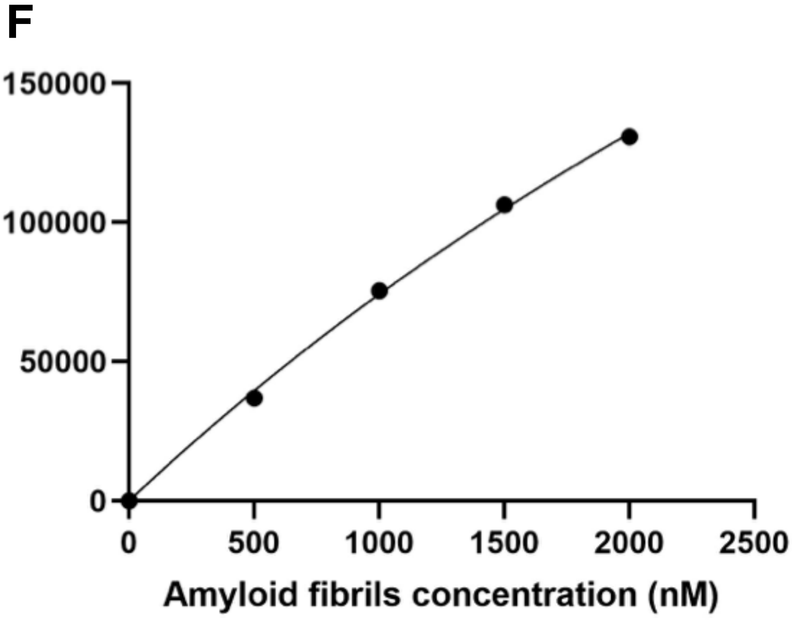

Figure S14. Dissociation constant determination, based on the variation (increasement) of the fluorescence intensity (**F**) of compound **6h** in the presence of increasing concentrations of fibrils of β-amyloid protein. λ_{ex}= 443 nm, λ_{em}= 592 nm.

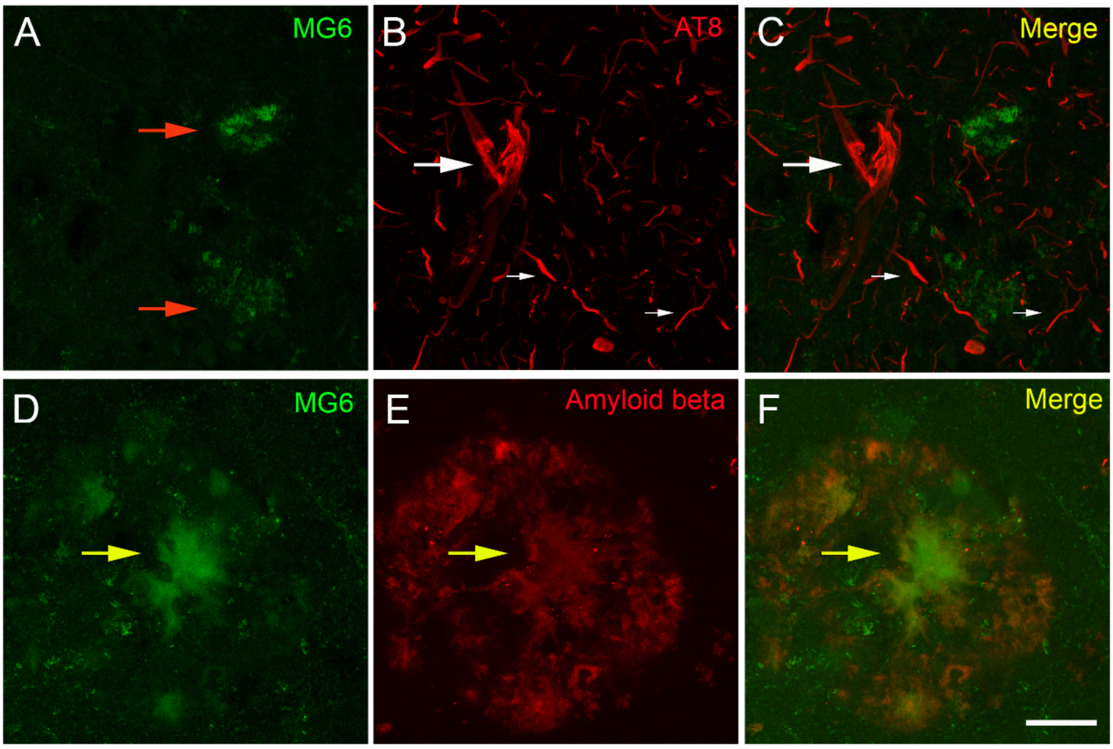

Figure S15. **Characterization of labelling by 6c in the temporal cortex of AD samples**. Z projection photomicrographs taken from confocal stacks show amyloid beta deposits labelled with MG6 in A and D. Labelling by AT8 antibody is shown in B, whereas labelling with amyloid beta antibody is shown in E. Small white arrows indicate some of the phosphorylated tau fibrils. Big white arrows indicate the paired helical filaments and big yellow arrows indicate the core of the amyloid beta plaque. White scale bar in F indicates 20 μm.

3. Additional Tables

Table S1: UV-Vis absorption properties of styrylquinoline derivatives **6**

**COMPOUND**	**λ_{max} (log ε)**
**6a**	255 (4.491), 356 (4.296), 441 (4.490)
**6b**	263 (3.533), 336 (3.573), 485 (3.773)
**6c**	290 (3.868), 335 (3.866), 455 (4.083)
**6d**	287 (4.098), 332 (4.025), 440 (4.282)
**6e**	292 (--), 319 (--), 435 (4.357)
**6f**	231 (4.194), 320 (3.943), 470 (4.083)
**6g**	270 (3.869), 348 (3.988), 502 (3.938)
**6h**	250 (4.212), 336 (3.896), 416 (4.019)

λ_{max}: maximum absorption UV-Vis wavelength in nm; ε: molar absorptivity in M^{-1} cm^{-1}

Table S2: Fluorescence excitation and emission wavelengths, in nm, of the compounds **6** in several solvents

CMPD.	*SOLVENT*				
	Hexane	*Dioxane*	*Ethanol*	*Acetonitrile*	*Water*
**6a**	- 385, 415, 535 (324)<br>- 520, 555 (sh) (465)	- 390, 410, 630 (331)<br>- 615 (470)	- 405, 430, 555 (332)<br>- 580 (467)	- 424, 565 (327)<br>- 590 (464)	- 445 (347)<br>- 570 (sh) (455)
**6b**	- 401, 426, 454, 565 (321)<br>- 615, 630 (488)	- 401, 426, 454, 625 (347)<br>- 625 (483)	- 433, 538 (339)<br>- 625 (480)	- 433, 538 (340)<br>- 630 (480)	- 412, 438, 464 (340)
**6c**	- 401, 426, 450, 540 (332)<br>- 550 (455)	- 401, 426, 450 (332)<br>- 650 (460)	- 410, 435, 550 (337)<br>- 560 (455)	- 410, 435, 550 (339)<br>- 560 (450)	- 435, 550 (337)<br>- 570 (429)
**6d**	- 401, 425, 451, 525 (337)<br>- 525 (438)	- 407, 433, 462 (sh), 625 (335)<br>- 628 (440)	- 413, 436, 539 (330)<br>- 549 (438)	- 410, 435, 535 (330)<br>- 545 (435)	- 410 (sh), 434, 545 (337)<br>- 575 (436)
**6e**	- 320, 330, 350 (sh), 525 (292)<br>- 520, 550 (sh), (459)	- 325, 335, 350 (sh) (292)<br>- 610 (457)	- 340, 535 (292)<br>- 540 (422)	- 550 (292)<br>- 560 (417)	- 450, 560 (305)<br>- 570 (447)
**6f**	- 385, 420, 540 (324)<br>- 545, 570 (sh) (481)	- 385, 405, 440, 620 (320)<br>- 630 (486)	- 405, 440, 540 (320)<br>- 555, (439)	- 335, 435, 560 (296)<br>- 560, (437)	535 (446)
**6g**	- 402, 407, 452, 575 (344)<br>- 575 (495)	- 407, 433, 461, 670 (344)<br>- 670 (496)	- 413, 437, 460, 560 (sh) (346)<br>- 579 (495)	- 413, 437, 460, (346)<br>- 575 (495)	- 393, 472 (349)<br>- 565 (483)
**6h**	- 385, 410, 575 (336)<br>- 590, 610 (sh) (442)	- 410, 430, 660 (339)<br>- 670 (443)	- 410, 430, 580 (339)<br>590 (419)	- 435, 590 (339)<br>- 600 (417)	- 320, 430 (262)<br>- 600 (447)

Excitation wavelengths in parenthesis
